# EBNA1BP2 (EBP2) promotes the progression of hepatocellular carcinoma through upregulating the expression of MCM8 and HMGB1

**DOI:** 10.1038/s41419-026-08671-8

**Published:** 2026-04-04

**Authors:** Ensi Ma, Hao Xing, Chao Sun, Quanbao Zhang, Conghuan Shen, Yangyang Zhan, Jianhua Li, Li Li, Hongyuan Xue, Ruidong Li, Fei Teng, Yifeng Tao

**Affiliations:** 1 Liver Transplantation Center, Department of General Surgery, Huashan Hospital & Institute of Organ Transplantation, Fudan University, Shanghai, China; 2 Department of Pharmacy, Shanghai Eastern Hepatobiliary Surgery Hospital, Navy Military Medical University, Shanghai, China; 3 Department of Intensive Care Unit, Huashan Hospital, Fudan University, Shanghai, China; 4 Department of Liver Surgery and Organ Transplantation, Changzheng Hospital, Naval Medical University, Shanghai, China

**Keywords:** Cancer therapy, Cell signalling

## Abstract

Primary liver cancer ranks among the most prevalent and refractory malignant tumors globally. This investigation delves into the role of Epstein-Barr virus nuclear antigen 2-binding protein (EBP2) in hepatocellular carcinoma (HCC). Significantly, EBP2 exhibits marked overexpression in HCC tissues, a finding that correlates with advanced tumor staging and unfavorable prognostic outcomes. In HCC cells, EBP2 silencing led to attenuated proliferation, enhanced apoptosis, and reduced migratory capacity, coupled with reversal of epithelial-mesenchymal transition (EMT). In vivo studies further demonstrated that EBP2 depletion potently suppressed tumor growth in xenograft models. Mechanistically, EBP2 interacts with CENPA to transcriptionally upregulate minichromosome maintenance protein family member 8 (MCM8), thereby stabilizing the MCM8/MCM9 complex and enhancing homologous recombination-mediated DNA repair. Functional rescue experiments revealed that MCM8 overexpression abrogated the suppressive effects of EBP2 knockdown on HCC cell proliferation and migration. In parallel, EBP2 regulates HMGB1 expression through the CENPA/YY1 transcriptional complex, thereby participating in the progression of HCC. Collectively, these findings highlight EBP2 as a crucial regulator of HCC progression via the dual axes-EBP2-CENPA-MCM8 and EBP2-CENPA/YY1-HMGB1, offering a promising therapeutic target for HCC intervention.

## Introduction

Primary liver cancer stands as one of the most prevalent and refractory malignant tumors worldwide. According to the GLOBOCAN cancer incidence and mortality data released by the International Agency for Research on Cancer (IARC), primary liver cancer ranks sixth in incidence among all malignant tumors and fourth in mortality [1, 2]. Pathologically, hepatocellular carcinoma (HCC) constitutes 85-90% of all primary liver cancer cases [3]. Hepatectomy and liver transplantation represent the primary modalities for radical treatment of HCC. However, the insidious onset of HCC poses a significant challenge for early-stage diagnosis. Consequently, the majority of patients present with advanced tumor stages or distant metastases at the time of diagnosis, with only 20–30% retaining eligibility for surgical resection [4, 5]. Characterized by pronounced inter-tumoral heterogeneity, rapid progression, inherent propensity for drug resistance, and high relapse/metastasis rates, HCC has yielded persistently poor prognoses for patients, with no fundamental improvements observed over recent decades [4]. In recent years, molecularly targeted therapies have emerged as promising therapeutic strategies for HCC. In 2006 and 2018, the US Food and Drug Administration (FDA) approved sorafenib and lenvatinib, respectively, as first-line targeted agents for HCC treatment [6, 7]. However, the clinical benefits of these agents remain suboptimal due to the inevitable development of drug resistance. Consequently, the identification of novel molecular targets represents a critical challenge in both basic and clinical HCC research, holding significant promise for advancing precise medicine approaches in HCC management [8].

Previous investigations have demonstrated that genes or proteins modulate carcinogenesis through diverse signaling pathways, playing pivotal roles in cancer diagnosis and therapy [9–12]. Epstein-Barr virus (EBV) relies on a single viral protein, Epstein-Barr nuclear antigen protein 1 (EBNA1), for its replication and maintenance within human cells [13, 14]. Conversely, EBNA1-binding protein 2 (EBNA1BP2, hereafter referred to as EBP2) is a host-derived protein identified via yeast two-hybrid screening that interacts with EBNA1 [15]. EBP2, a highly conserved protein with a molecular weight of 35 kDa, has been demonstrated to mediate the interaction between EBNA1 and metaphase chromosomes during cell division, thereby playing a critical role in the partitioning of EBV episomes during mitosis [16]. Consistently, EBP2 silencing not only impairs human cell proliferation but also markedly diminishes EBNA1-mediated association of EBV viral plasmids with mitotic chromosomes [17, 18]. Notably, while EBNA1 has been established as a critical mediator of EBV-induced malignancies, the role of EBP2 in carcinogenesis has been scarcely investigated, with mechanistic insights remaining elusive [19–21]. Previous studies have revealed that stable expression of EBP2 in cells is associated with both transcriptional and translational upregulation of the oncogenic cyclin E1, heightened p21 expression, and enhanced serine-15 phosphorylation of p53. These findings suggest an intrinsic link between EBP2 overexpression and tumorigenic progression [22].

This study aims to systematically investigate the role and underlying mechanisms of EBP2 in HCC progression, as well as its potential as a prognostic biomarker and therapeutic target. Specifically, we will characterize EBP2 expression patterns in HCC tissues and correlate them with clinical parameters and patient outcomes. The functional consequences of EBP2 depletion on HCC cell proliferation, apoptosis, migration, and in vivo tumorigenesis will be systematically evaluated. Mechanistically, this study aims to dissect the downstream signaling cascades through which EBP2 modulates HCC progression, with a specific focus on its transcriptional regulation of minichromosome maintenance protein family member 8 (MCM8) and HMGB1 *via* the chromatin-associated factor CENPA. By establishing the functional and mechanistic interrelationship between EBP2 and MCM8/HMGB1, this investigation seeks to unravel the molecular determinants of HCC pathogenesis and identify actionable targets for therapeutic intervention.

## Materials and methods

### Immunohistochemistry (IHC) analysis

A tissue microarray of HCC, comprising 80 HCC tissues and 71 matched normal liver tissues, which were collected from Huashan Hospital, Fudan University, was employed to assess EBP2 expression in human HCC tissues. Initially, paraffin-embedded tissue sections were subjected to dewaxing with xylene, followed by rehydration through a gradient series of alcohol solutions. Antigen retrieval was performed using sodium citrate buffer. The specimens were subsequently washed three times with 1× phosphate-buffered saline (PBS) containing 0.1% Tween20 (Beyotime, China). Endogenous peroxidase activity was blocked by incubation with 3% H_2_O_2_ for 5 min. Following another wash with PBS, the tissues were subjected to blocking with 5% serum for 15 min, followed by incubation with the primary antibody at 37 °C for 1 h. After subsequent PBS washes, the tissues were incubated with a secondary antibody at 37 °C for 1 h. The samples were stained with 3,3’-diaminobenzidine (DAB) solution in the dark, followed by counterstaining with hematoxylin. The antibodies utilized in this study are detailed in Table S1. Immunohistochemical (IHC) scoring was performed based on the percentage of positive cells: 0–25% = 1 point; 26–50% = 2 points; 51–75% = 3 points; ≥76% = 4 points. Staining intensity was evaluated as follows: no staining = 0 points; light yellow = 1 point; pale brown = 2 points; dark brown = 3 points. The total score for each sample was calculated by multiplying the percentage score by the staining intensity score. All procedures involving human participants were approved by the Institutional Review Board (IRB) of Huashan Hospital, Fudan University (Approval No. [FE23194R]). The study was conducted in accordance with the Declaration of Helsinki, and informed consent was obtained from all participants.

### Bioinformatics analysis

To investigate the expression of EBP2 and its downstream target MCM8 in the context of HCC progression, gene expression profiles from The Cancer Genome Atlas Program (TCGA) and Gene Expression Omnibus (GEO) databases were systematically analyzed. RNA-seq data of HCC and normal liver tissue samples from TCGA (https://portal.gdc.cancer.gov/) and GEO (http://www.ncbi.nlm.nih.gov/geo/) were processed using the DESeq2 package in R for normalization and differential expression analysis. Human HCC datasets from cbioportal (https://www.cbioportal.org/) were integrated into the analysis to enhance statistical power. RNA-seq counts were normalized and log-transformed using the log2 (x + 1) transformation, where x denotes the RSEM-estimated gene expression values, to facilitate inter-sample comparison and downstream bioinformatics analyses.

### Cell culture

The normal hepatic cell line LX-2, along with HCC cell lines HCCLM3, Hep3B, HepG2, and Huh-7, were procured from BNCC Co., Ltd (Henan, China). HCCLM3 and Huh-7 cells were cultivated in 90% Dulbecco’s Modified Eagle Medium (DMEM-H) supplemented with 10% fetal bovine serum (FBS). HepG2 and Hep3B cells were grown in 90% Eagle’s Minimum Essential Medium (EMEM) containing 10% FBS. LX-2 cells were maintained in 90% Roswell Park Memorial Institute Medium (RPMI-1640) with 10% FBS.

### Construction of cell models

Lentiviral vectors were engineered to modulate EBP2 or MCM8 expression in HCC cells. Target-specific sequences for EBP2 (5’-ACGAACAAGAGAGAAGATGAA-3’; 5’-TCGACAGAAGCTGCAGACTAA-3’; 5’-GCCGAAGAAGGCCGTGAACGA-3’) and MCM8 (5’-TGGCAATACATCAGGTGTTAA-3’; 5’-CTGGAATTGTCAAAGTCTCAA-3’; 5’-AGGCAGCTGGAATCTTTGATT-3’) were designed. Single-stranded DNA oligonucleotides were synthesized, annealed, and ligated to form double-stranded DNA fragments with cohesive ends for subsequent cloning into lentiviral backbones. Linear vectors were prepared in a 50 μL reaction system following the manufacturer’s protocol from New England Biolabs (NEB, USA). Double-stranded DNA fragments were ligated into the linear vectors using 20 μL reaction mixtures containing T4 DNA Ligase (Fermentas, USA), as per the supplier’s instructions. The ligation products were transformed into competent *Escherichia coli* cells, which were then selected on agar plates supplemented with ampicillin. Positive colonies were identified via quantitative polymerase chain reaction (PCR), and plasmids were extracted using an EndoFree Maxi Plasmid Kit (TIANGEN, China). Lentivirus packaging was performed using the BR-V108 plasmid in conjunction with auxiliary plasmids pMD2.G and pSPAX2, obtained from Shanghai Yibeirui Biomedical Science and Technology Co., Ltd (Shanghai, China). Human HCC cells were seeded into 6-well plates at a density of 2 × 10^5^ cells per well and infected with lentiviral plasmids. The cells were incubated at 37 °C with a 5% CO_2_ atmosphere. Following an 18 h incubation, the culture medium was replaced with fresh medium. Fluorescence expression and infection efficiency were evaluated using a microscope (OLYMPUS, Japan).

### Quantitative polymerase chain reaction (qPCR)

Total RNA was extracted from cells using the Trizol method (Sigma, USA) according to the manufacturer’s protocol. Following centrifugation, RNase-free H_2_O was added to the cell pellets for lysis. The concentration and purity of extracted RNA were determined using a Nanodrop 100 spectrophotometer (Thermo, Germany). Complementary DNA (cDNA) was synthesized from 1 μg of total RNA using the Hiscript QRT supermix for qPCR (+gDNA WIPER) (Vazyme, China). The specific primers used for PCR amplification of target genes are detailed in Table S2.

### Western blotting (WB)

Cells were lysed on ice at 4 °C, and total protein concentration was quantified using a BCA Protein Assay Kit (HyClone-Pierce, USA). Equal amounts (20 μg) of protein samples were separated by SDS-PAGE and electrotransferred onto polyvinylidene fluoride (PVDF) membranes using a protein transfer system (Tanon, China). The PVDF membranes were blocked with 5% skim milk in TBST and then incubated with primary antibodies overnight at 4 °C. Following extensive washing with TBST, the membranes were probed with corresponding secondary antibodies for 1 h at room temperature. Protein bands were detected using Immobilon Western Chemiluminescent HRP Substrate (Millipore, USA) and visualized with a chemiluminescence system (GE, USA). The primary and secondary antibodies used in this study are listed in Table S1.

### Cell counting kit-8 (CCK-8) assay

Lentivirus-transduced cells were seeded into 96-well plates at a density of 2.5 × 10^3^ cells per well. Following incubation, 10 μL of CCK-8 solution (Sigma, USA) was added to each well. After 4 h of incubation at 37 °C, the optical density (OD) at 450 nm was measured using a microplate reader (Tecan Infinite, Switzerland).

### Flow cytometry

For apoptosis assessment, cells were detached, centrifuged, and washed with 1× binding buffer. Cell suspensions were incubated with 5 μL of annexin V-APC conjugate, followed by centrifugation and resuspension in 100 μL of 1× binding buffer. Propidium iodide (PI, 5 μL) was added for counterstaining, and apoptosis was quantified using a flow cytometer (Millipore, USA). For cell cycle analysis, cells were trypsinized, centrifuged, and washed with PBS prior to fixation in 70% ethanol. Fixed cells were stained with a solution (40× PI: 100× RNase: 1×PBS = 25: 10: 1000) and analyzed by a Millipore flow cytometer (USA).

### Colony formation

Lentivirus-transduced cells were seeded into 6-well plates at a density of 1 × 10^3^ cells per well. Following a 14-day incubation period, the cells were washed with PBS, fixed with 4% paraformaldehyde, and stained with crystal violet solution (Beyotime, China). The stained colonies were rinsed with distilled water (ddH_2_O) and documented using bright-field imaging.

### Wound healing assays

Lentivirus-transduced HCCLM3 and Hep3B cells were seeded into 96-well plates at a density of 5 × 10^4^ cells per well. A standardized scratch wound was created using a sterile pipette tip, and cellular debris was removed by washing with serum-free medium. Cells were subsequently cultured in medium supplemented with 0.5% FBS at 37 °C with a 5% CO_2_ atmosphere. Wound healing was monitored over time using a Cellomics system (Thermo, USA), and the rate of wound healing was analyzed using proprietary software.

### Transwell assay

Lentivirus-transduced HCCLM3 and Hep3B cells were seeded into fibronectin-coated Transwell insert (Costar, MA) at a density of 100 μL of serum-free medium. The lower chamber was filled with medium containing 10% FBS as a chemoattractant. Following incubation at 37 °C the membrane was fixed with 4% paraformaldehyde, stained with 1% crystal violet, and quantified by counting cells in three random high-power fields (HPF) under an optical microscope.

### Animal xenograft assay

Ten 4-week-old female BALB/c-nude specific pathogen-free (SPF) mice were procured from GemPharmatech Co., Ltd (Jiangsu, China) and randomized and allocated into two groups: the control group (sh-Ctrl) and the EBP2 knockdown group (sh-EBP2), with 5 mice per group (*n* = 5). The mice were maintained under standard laboratory conditions: temperature at 22 ± 2 °C, relative humidity of 55 ± 5%, and a 12-h light/dark cycle. Sterilized food and water were provided *ad libitum*. HCCLM3 cells with stable EBP2 knockdown or control shRNA were subcutaneously injected into the flanks of nude mice at a concentration of 1 × 10^7^ cells/mL (0.2 mL per injection). Tumor volumes were measured every 3 days using the formula: π/6 × length × width^2^. Prior to reaching the humane endpoint, tumor fluorescence intensity was evaluated using an IVIS Spectrum imaging system (Perkin Elmer). Mice were euthanized via intraperitoneal injection of pentobarbital sodium (120 mg/kg), and tumors were excised, weighed, and processed for IHC analysis of Ki67 expression. The primary antibodies used in this study are detailed in Table S1. All animal experiments were approved by the Institutional Animal Care and Use Committee (IACUC) of Fudan University (Approval No. [2023-HSYY-293-JZS]). All procedures were performed in accordance with the institutional and national guidelines for the care and use of laboratory animals.

### Gene chip assay

Affymetrix human GeneChip PrimeView microarray analysis was conducted to profile differential gene expression in HCCLM3 cells transfected with sh-EBP2 or sh-Ctrl lentiviruses. Raw intensity data were processed using Welch’s t-test with Benjamini-Hochberg false discovery rate (FDR) correction, and genes with an absolute |Fold Change|≥1.3 and FDR < 0.05 were identified as significantly differentially expressed. Pathway enrichment and biological annotation analyses were performed using Ingenuity Pathway Analysis (IPA) to characterize biological processes and signaling networks associated with EBP2 knockdown.

### Chromatin immunoprecipitation (ChIP) assays

ChIP assays were performed using the SimpleChIP Enzymatic Chromatin IP Kit (Agarose Beads) (#9002, CST, USA) following the manufacturer’s protocol. HCCLM3 and Hep3B cells were cross-linked with formaldehyde to stabilize protein-DNA interactions, followed by lysis and sonication to fragment chromatin into 200–1000 bp DNA fragments. Immunoprecipitation of CENPA-protein-DNA complexes was achieved using antibodies specific to CENPA. Complexes were dissociated by adding SDS, and DNA was purified using spin columns. Quantitative PCR was performed to quantify DNA fragments, with specific primers targeting the MCM8 promoter region to assess CENPA binding affinity.

### Double luciferase reporter assay

A reaction mixture was prepared containing 10 μL of DMEM, 0.16 μg MCM8-WT or MCM8-Mut promoter reporter plasmids, and 5 pmol of transcription factor CENPA expression vector or negative control (NC) plasmid. This mixture was combined with a separate solution consisting of 10 μL DMEM and 0.3 μL of Lipo-3000 transfection reagent (0.8 mg/mL), followed by incubation at room temperature for 20 min to allow formation of transfection complexes. The resulting complexes were added to HCCLM3 or Hep3B cells and cultured at 37 °C in a 5% CO_2_ incubator. After 48 h, cells were harvested, and luciferase activity was measured using the Dual-Luciferase system assay kit (Promega) according to the manufacturer’s protocol.

### Co-immunoprecipitation (Co-IP) assay

Protein extracts (1.0 mg) were incubated overnight at 4 °C with specific antibodies, followed by a 2-h incubation with 20 μL protein A/G magnetic beads at the same temperature to form protein-antibody-bead complexes. The complexes were washed extensively with ice-cold lysis buffer, separated by SDS-PAGE, and transferred to PVDF membranes. WB analysis was performed using anti-MCM8 antibodies, and protein bands were detected by chemiluminescence with a dedicated imaging system. The primary and secondary antibodies used in this study are detailed in Table S1.

### Statistical analysis

Data are expressed as mean ± standard deviation (SD) from at least three independent experiments, analyzed using GraphPad Prism 8.0 software (GraphPad Software Inc., USA). For intergroup comparisons, a paired two-tailed Student’s *t* test was employed for analyses involving two groups, where appropriate, either one-way or two-way repeated measures (ANOVA) was conducted, followed by Bonferroni’s post hoc test for multiple comparisons. The chi-square test was utilized to examine the association between the expression levels of target genes and patients’ clinicopathological parameters. Non-parametric analyses were performed using the Mann–Whitney U test when data did not conform to normal distribution. Pearson correlation analysis was used to assess linear relationships between variables. Statistical significance was defined as a *P* < 0.05.

## Results

### Overexpression of EBP2 in HCC

**Fig. 1:**
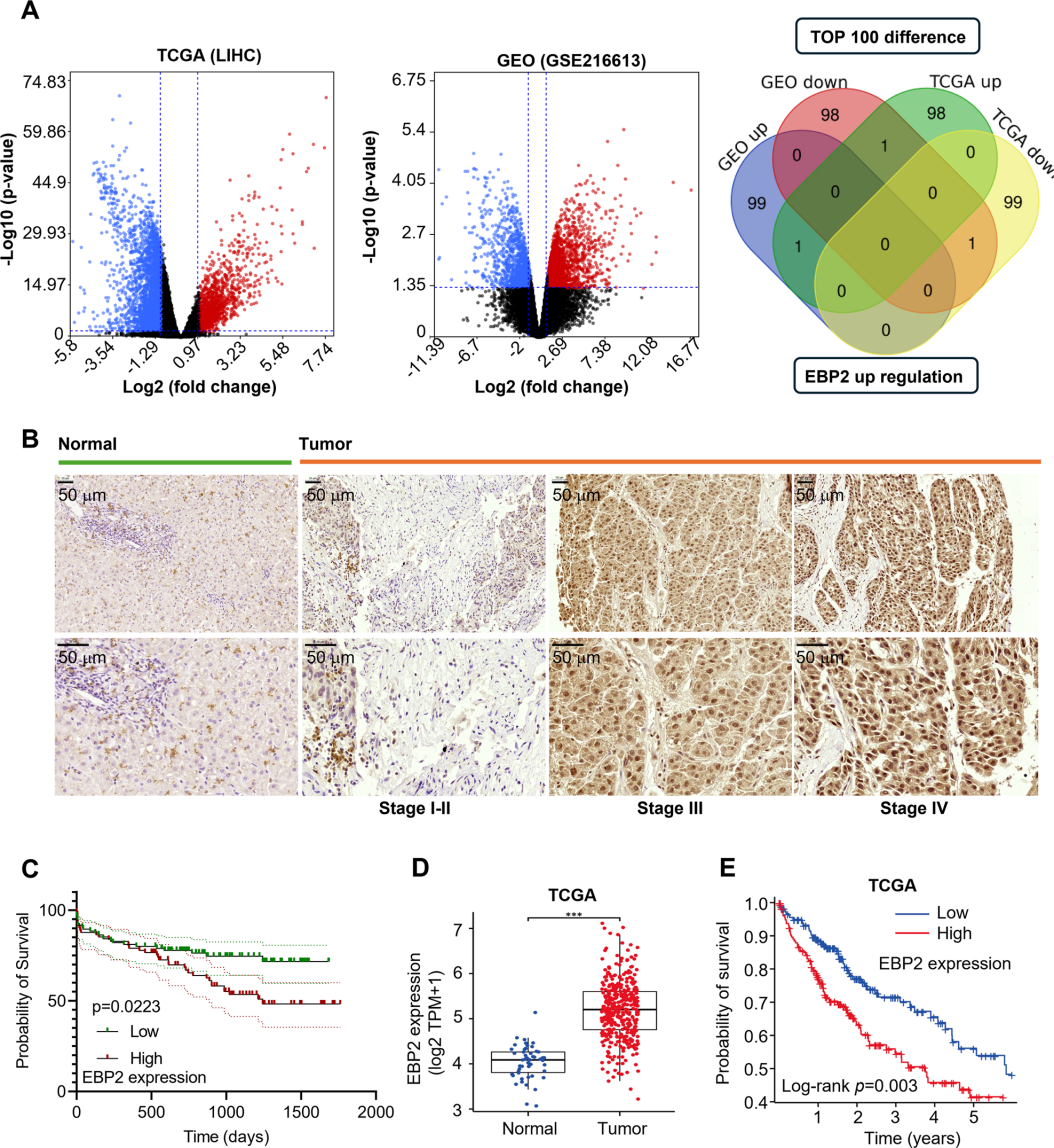
EBP2 expression and its prognostic significance in HCC. **A** The volcano plot and Venn Diagram of differentially expressed mRNAs in HCC tissues based on the TCGA-LIHC and GEO database, the results revealed that EBP2 was significantly upregulated in HCC. **B** Immunohistochemical analysis of EBP2 expression in normal liver tissues and HCC using a tissue microarray. EBP2 expression is significantly higher in HCC tissues compared to normal tissues, with a positive correlation between EBP2 levels and tumor stage. Scale bar = 50 μm. **C** Kaplan–Meier survival analysis of HCC patients based on EBP2 expression from the tissue microarray cohort. High EBP2 expression is associated with worse overall survival. **D** Analysis of EBP2 expression in HCC and normal tissues using TCGA-LIHC data. EBP2 is significantly upregulated in HCC tissues. **E** Kaplan–Meier survival analysis of HCC patients based on TCGA-LIHC data. High EBP2 expression correlates with poorer prognosis. Data were shown as mean ± SD (*n* ≥ 3). *** *P* < 0.001.

To identify differentially expressed genes (DEGs) in HCC, publicly available transcriptomic datasets were downloaded from The Cancer Genome Atlas (TCGA-LIHC) and the GEO database. Volcano plot analysis of TCGA-LIHC data revealed 1285 upregulated and 3401 downregulated mRNAs in HCC tissues relative to normal controls (Fig. 1A left). Concurrently, differential expression analysis for the GEO database (GSE216613) identified 3256 DEGs, including 1154 down-regulated and 2102 up-regulated mRNAs in HCC tissues (Fig. 1A middle). Subsequently, the top 100 significantly upregulated and downregulated genes were selected from each dataset for Venn diagram analysis to identify intersecting genes. Notably, only one upregulated gene, EBP2, was shared between the two gene sets (Fig. 1A right). Given the limited research on EBP2 in cancer, this gene was selected as the primary focus of the study. IHC analysis was performed using a tissue microarray containing 80 HCC tissues and 71 matched normal liver tissues to evaluate EBP2 expression. The results showed that EBP2 expression was significantly higher in HCC tissues than in normal tissues (Fig. 1B). Statistical analysis validated these findings, demonstrating significant upregulation of EBP2 in tumor tissues (Table 1). Correlation analyses further revealed a positive association between EBP2 expression and multiple clinicopathological parameters of HCC, including tumor stage and T-cell infiltration (Table 2). Notably, EBP2 expression levels exhibited a stage-dependent increase, with higher expression observed in advanced-stage HCC (Fig. 1B and Tables S2; S3). Kaplan-Meier survival analysis revealed that high EBP2 expression was associated with poor prognosis in HCC patients (Fig. 1C). To validate these findings, RNA sequencing data from the TCGA-LIHC database were analyzed to compare EBP2 expression between HCC and normal liver tissues. Consistent with IHC results, EBP2 mRNA levels were significantly upregulated in HCC tissues relative to normal controls (Fig. 1D). Kaplan-Meier survival analysis using TCGA-LIHC data further corroborated that high EBP2 expression was associated with poorer overall survival in HCC patients (Fig. 1E). To evaluate whether EBP2 expression could serve as an independent prognostic factor, multivariate Cox proportional hazards regression analysis was performed using clinical and transcriptomic data from TCGA-LIHC. The results demonstrated that EBP2 expression was an independent predictor of overall survival, after adjusting for other clinicopathological parameters (Table 3). These findings highlight EBP2 as a potential prognostic biomarker and therapy target for HCC.

**Table 1:** Expression patterns of EBP2 in HCC tissues and normal tissues were revealed in immunohistochemistry analysis.

EBP2 expression	Tumor tissue		Normal tissue	
	Cases	Percentage	Cases	Percentage
Low	45	56.2%	61	85.9%
High	35	43.8%	10	14.1%

*P* < 0.001.

**Table 2:** Relationship between EBP2 expression and tumor characteristics in patients with HCC.

Features	No. of patients	EBP2 expression		*P* value
		low	high	
All patients	80	45	35	
Age (years)				0.126
≤60	42	20	22	
>60	37	24	13	
Gender				0.884
Male	66	37	29	
Female	13	7	6	
Grade				0.745
II	40	23	17	
III	39	21	18	
Tumor size				0.094
≤6 cm	40	26	14	
>6 cm	39	18	21	
Stage				0.006*
I	24	19	5	
II	37	18	19	
III	17	7	10	
IV	1	0	1	
T Infiltrate				0.014*
T1	24	19	5	
T2	38	18	20	
T3	6	1	5	
T4	11	6	5

**Table 3:** Multivariate Cox regression analysis of EBP2 expression in TCGA-LIHC data.

Characteristics	HR (95% CI) Univariate analysis	*P* value Univariate analysis	HR (95% CI) Multivariate analysis	*P* value Multivariate analysis
**Pathologic T stage**				
T1	Reference		Reference	
T2	1.431 (0.902–2.268)	0.128	1.287 (0.804–2.060)	0.293
T3	2.674 (1.761–4.060)	<0.001	2.488 (1.629–3.798)	<0.001
T4	5.386 (2.690–10.784)	<0.001	4.826 (2.394–9.729)	<0.001
**Pathologic N stage**				
N0	Reference			
N1	2.029 (0.497–8.281)	0.324		
**Pathologic M stage**				
M0	Reference			
M1	4.077 (1.281–12.973)	0.017		
**Histologic grade**				
G1	Reference			
G2	1.162 (0.686–1.969)	0.576		
G3	1.185 (0.683–2.057)	0.545		
G4	1.681 (0.621–4.549)	0.307		
**AFP (ng/ml)**				
≤400	Reference			
å 400	1.075 (0.658–1.759)	0.772		
**EBP2**				
Low	Reference		Reference	
High	1.692 (1.192–2.402)	0.003	1.480 (1.033–2.121)	0.033

### EBP2 plays a tumor-promoting role in HCC in vitro and in vivo

**Fig. 2:**
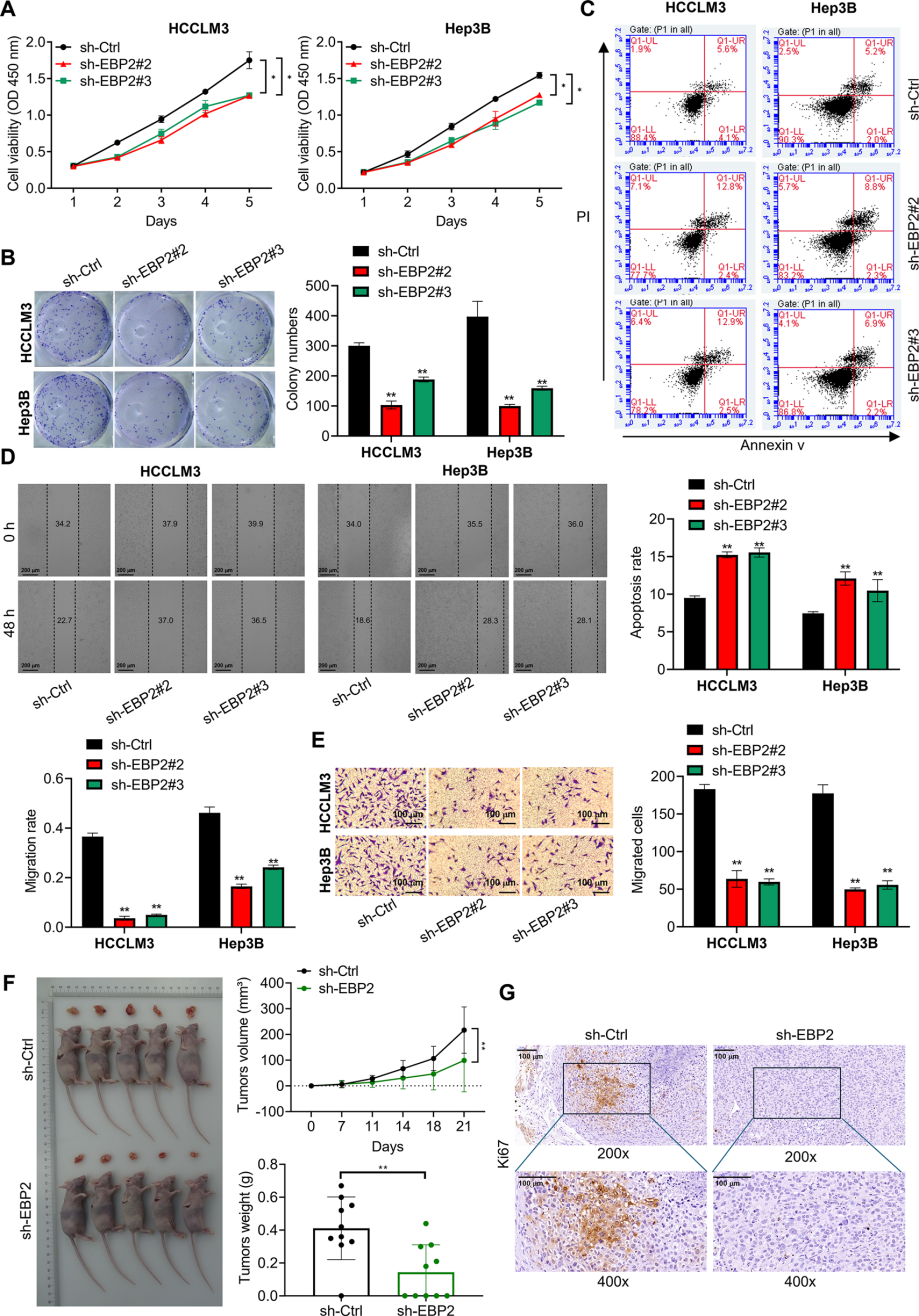
The effects of EBP2 knockdown on HCC cell proliferation, apoptosis, migration and tumor growth. **A** CCK-8 assay showing that EBP2 knockdown significantly inhibits the proliferation of HCCLM3 and Hep3B cells. **B** Colony formation assay indicating a reduced number of colonies in EBP2 knockdown HCCLM3 and Hep3B cells. **C** Flow cytometry analysis demonstrating increased apoptosis in HCCLM3 and Hep3B cells upon EBP2 knockdown. **D** Wound-healing assay revealing decreased migration in EBP2 knockdown HCCLM3 and Hep3B cells. Scale bar = 200 μm. **E** Transwell migration assay showing a significant decrease in the migration of HCCLM3 and Hep3B cells following EBP2 knockdown. Scale bar = 100 μm. **F** Tumor volume and weights measurements over time showing that EBP2 knockdown (sh-EBP2) significantly slows tumor growth compared to the control (sh-Ctrl). **G** Immunohistochemical staining for Ki67 in tumor sections, showing reduced Ki67 expression in sh-EBP2 tumors, indicative of decreased cell proliferation. Scale bar = 100 μm. Data were shown as mean ± SD (*n* ≥ 3). *P < 0.05, ** *P* < 0.01.

To characterize the basal expression profile of EBP2, qPCR was performed in normal hepatic stellate cells (LX-2) and multiple HCC cell lines (HCCLM3, Hep3B, HepG2, and Huh-7). qPCR analysis revealed that EBP2 mRNA expression was significantly upregulated in all HCC cell lines relative to LX-2 cells, with the highest expression levels detected in HCCLM3 and Hep3B cells (Fig. S1A). Consequently, these two cell lines were chosen for subsequent experiments. The knockdown efficiencies of three distinct short hairpin RNAs (sh-RNAs) targeting EBP2 (sh-EBP2#1#2#3) were evaluated by qPCR. Both sh-EBP2#2 and sh-EBP2#3 exhibited significantly higher knockdown efficiency than sh-EBP2#1, prompting their selection for further investigations (Fig. S1B). Validation of EBP2 knockdown in HCCLM3 and Hep3B cells via qPCR and WB analysis confirmed the successful reduction of EBP2 expression by sh-EBP2#2 and sh-EBP2#3 (Fig. S1C, D). The effect of EBP2 on HCC cell proliferation was evaluated using the CCK8 assay. Results showed that EBP2 knockdown significantly inhibited the proliferation of both HCCLM3 and Hep3B cells compared to control groups (Fig. 2A). Concurrently, colony formation assays demonstrated a marked reduction in the number of colonies formed by EBP2-depleted HCCLM3 and Hep3B cells, further confirming that EBP2 knockdown suppressed cell proliferation and clonogenic survival (Fig. 2B). Flow cytometry was used to evaluate the apoptosis rate in HCC cells after EBP2 knockdown. The results demonstrated a significant increase in apoptosis in both HCCLM3 and Hep3B cells with decreased EBP2 expression, indicating that EBP2 is involved in the survival of HCC cells (Fig. 2C). The migratory ability of EBP2-knockdown HCC cells was assessed by wound-healing and Transwell migration assays. Notably, EBP2 knockdown significantly impaired the migratory capacity of HCCLM3 and Hep3B cells, as demonstrated by reduced wound closure rates in scratch assays and decreased cell migration in Transwell assays (Fig. 2D, E). To investigate the molecular mechanisms underlying these phenotypic alterations, WB analysis was conducted to assess the expression levels of epithelial-mesenchymal transition (EMT)-associated proteins. The results showed that EBP2 knockdown resulted in upregulation of the epithelial marker E-cadherin and downregulation of the mesenchymal markers N-cadherin, Vimentin, and Snail, suggesting a reversal of EMT in HCC cells (Fig. S2). To further validate the role of EBP2 in vivo, a nude mouse xenograft model was established by subcutaneously inoculating HCCLM3 cells stably transfected with sh-EBP2 or scrambled control (sh-Ctrl) lentiviruses. Xenograft tumor analysis revealed that both tumor volume and weight were significantly reduced in the sh-EBP2 group compared to the control group (Fig. 2F), demonstrating that EBP2 promotes in vivo tumorigenicity of HCC cells. IHC staining further showed that EBP2 depletion markedly decreased the positive expression rate of Ki67 (Fig. 2G), indicating suppressed cell proliferation following EBP2 knockdown. Collectively, these in vivo findings confirm that EBP2 downregulation inhibits HCC tumor growth. Taken together, these results establish EBP2 as a critical tumor-promoting factor in HCC both in vitro and in vivo.

### Exploration of downstream mechanisms of EBP2 regulation in HCC progression

**Fig. 3:**
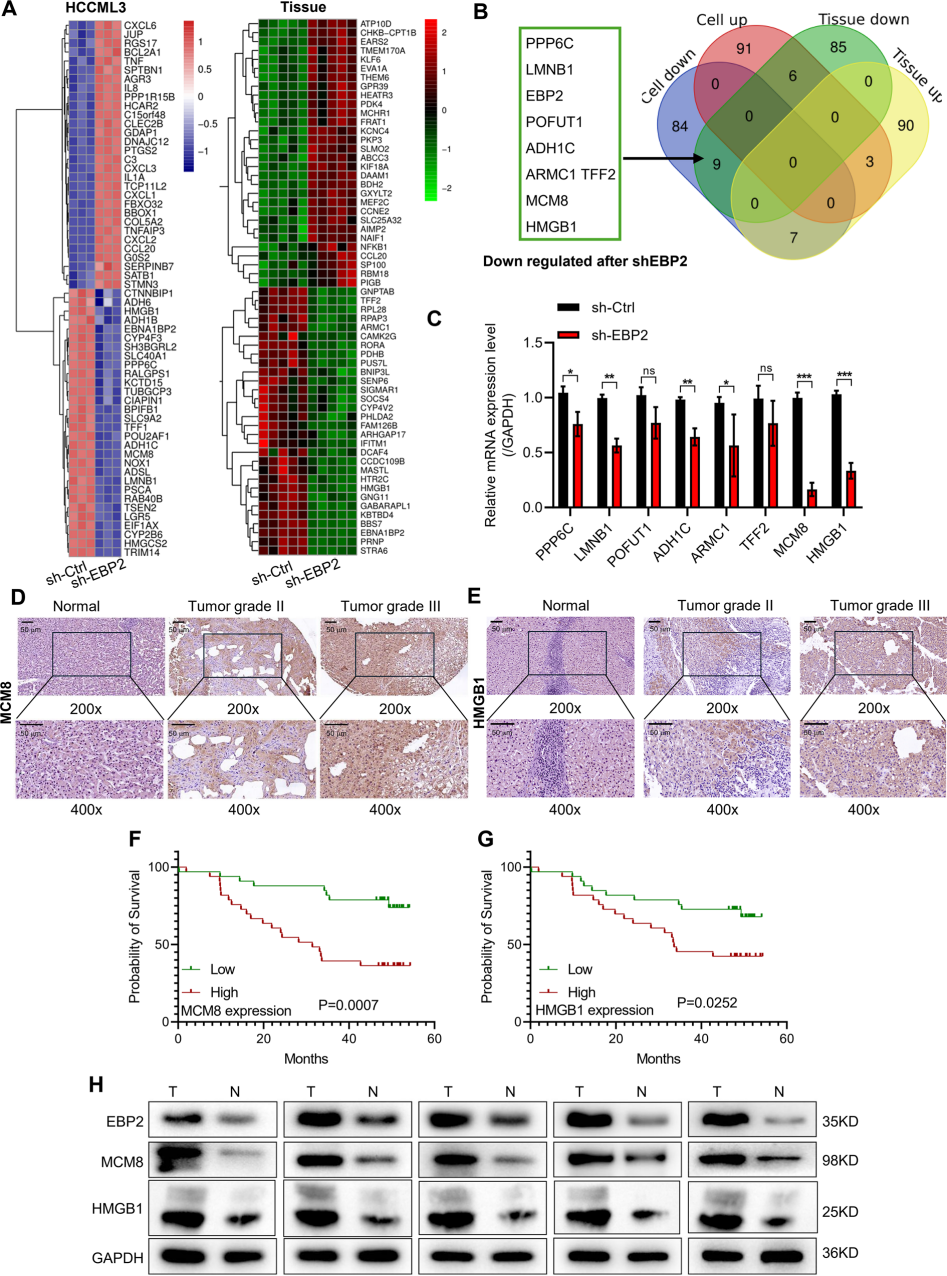
EBP2 regulates HCC progression through MCM8 and HMGB1. **A** Heatmap of differentially expressed genes in HCCLM3 cells and HCC tissues transfected with sh-EBP2 versus sh-Ctrl. **B** Venn diagram intersection gene analysis was used to screen for common genes; the results showed that there were nine duplicated genes in the above 2 collections (cell down and tissue down). **C** qPCR analysis was performed in HCCLM3 to detect the expression levels of several mRNAs screened during EBP2 knockdown. The results confirmed the significant downregulation of several genes, including LMNB1, ADH1C, ARMC1, MCM8, and HMGB1. **D** IHC staining of MCM8 in tumor and normal tissues, demonstrating higher expression in tumors and correlation with malignancy. Scale bar = 50 μm. **E** IHC staining of HMGB1 in tumor and normal tissues, demonstrating higher expression in tumors and correlation with malignancy. Scale bar = 50 μm. **F** Survival analysis based on tissue microarray data, showing lower survival rates in patients with high MCM8 expression. **G** Survival analysis based on tissue microarray data, showing lower survival rates in patients with high HMGB1 expression. **H** WB analysis confirming high expression of EBP2, MCM8 and HMGB1 in tumor tissues compared to normal tissues. Data were shown as mean ± SD (*n* ≥ 3). *P < 0.05, ** *P* < 0.01, *** *P* < 0.001.

To investigate the downstream molecular mechanisms through which EBP2 regulates HCC progression, gene expression profiling was conducted using microarray analysis on HCCLM3 cells and mouse tumorous tissues transfected with sh-EBP2 or sh-Ctrl lentiviruses. The heatmap in Fig. 3A and the volcano plot in Fig. S3A illustrate the differentially expressed genes between the two groups, demonstrating substantial transcriptional changes following EBP2 knockdown. Venn diagram analysis of intersecting gene sets identified nine overlapping genes between the two datasets (genes downregulated in both HCCLM3 cells and tumor tissues) (Fig. 3B). Subsequent PCR analysis was performed to assess the mRNA expression levels of eight selected genes in HCCLM3 cells transfected with sh-Ctrl or sh-EBP2 lentiviruses, confirming significant downregulation of LMNB1, ADH1C, ARMC1, MCM8, and HMGB1 (Fig. 3C). WB analysis further validated the protein expression of these genes, demonstrating that EBP2 significantly modulated the expression of HMGB1 and MCM8 (Fig. S3B). Notably, MCM8 and HMGB1 were consistently downregulated in both HCCLM3 cells and mouse tumor tissues upon EBP2 knockdown, supporting their selection as robust downstream effectors. Using the TCGA-LIHC database, the correlation between EBP2 expression and that of MCM8 and HMGB1 was analyzed. The results showed a significant positive correlation between EBP2 and both MCM8 and HMGB1 (Fig. S3C). Further analysis of TCGA data revealed that MCM8 and HMGB1 expression was significantly higher in tumor tissues than in normal tissues (Fig. S3D). Survival analysis also indicated that high MCM8 and HMGB1 expression was associated with poor prognosis in HCC patients (Fig. S3E). To validate the expression profile of MCM8 and HMGB1 in clinical specimens, a tissue microarray comprising 68 tumor tissues and 83 normal tissues was utilized. IHC staining revealed that MCM8 expression was significantly upregulated in tumor tissues and exhibited a positive correlation with tumor malignancy (Fig. 3D, E). Statistical analysis (Tables S4–S9) further confirmed these observations, demonstrating that elevated MCM8 and HMGB1 expression was associated with higher tumor grades. Survival analysis using tissue microarray data further confirmed that high MCM8 and HMGB1 expression was associated with reduced survival rates in HCC patients (Fig. 3F, G). The significant upregulation of MCM8 and HMGB1 in HCC and their correlation with poor patient survival underscore their potential as both biomarkers and therapeutic targets. Furthermore, WB analysis of paired tumor and normal tissues from five HCC patients demonstrated elevated expression of EBP2, MCM8 and HMGB1 in tumor tissues (Fig. 3H). Moreover, quantitative analysis revealed that MCM8 and HMGB1 mRNA expression was significantly upregulated in HCC cell lines relative to normal hepatic stellate cells (Fig. S3F, G).

### EBP2 may regulate MCM8 transcription *via* CENPA

**Fig. 4:**
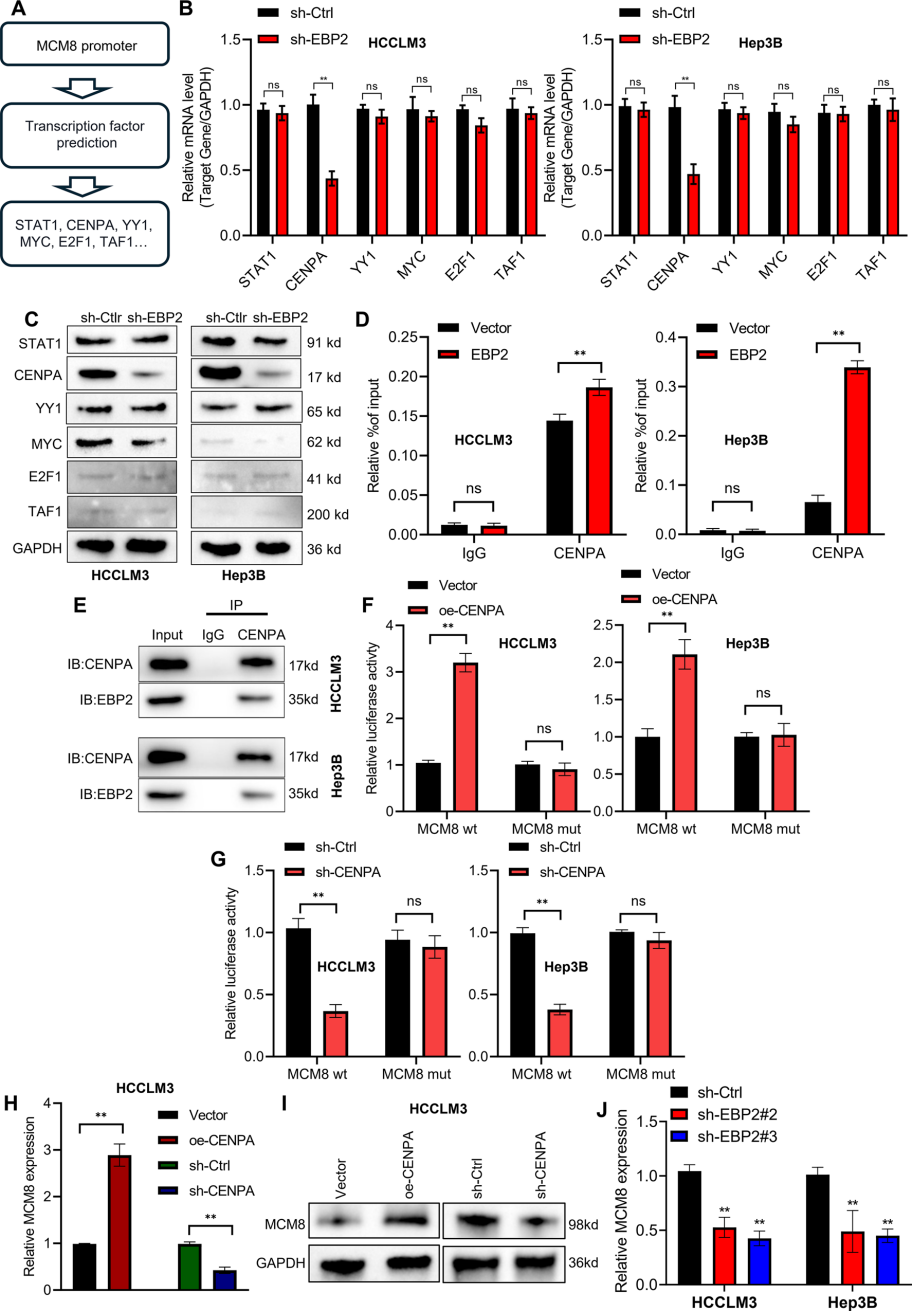
EBP2 enhances MCM8 transcription through interaction with CENPA. **A** Using bioinformatics analysis to predict potential transcription factors binding to the promoter region of MCM8, the results showed that EBP2 might regulate MCM8 expression through EBP2. **B** qPCR results showed that the CENPA mRNA expression was reduced in HCCLM3 and Hep3B cells transfected with sh-EBP2. **C** WB analysis confirming that CENPA was significantly regulated by EBP2. **D** ChIP assay demonstrating increased binding of CENPA to the MCM8 promoter in EBP2 overexpression cells compared to the control group. Anti-CENPA antibodies were used for IP, with IgG as a negative control. Scale bar = 30 μm. **E** co-IP confirming the protein-protein interaction between EBP2 and CENPA. **F** Dual-luciferase reporter assay showing that overexpression of CENPA enhances the activity of the MCM8 promoter, while mutation of the predicted CENPA binding sites abolishes this effect. **G** Dual-luciferase reporter assay showing that CENPA knockdown reduces the activity of the MCM8 promoter, while mutation of the predicted CENPA binding sites abolishes this effect. **H**, **I** qPCR and WB analysis confirming that MCM8 was significantly regulated by CENPA. **J** qPCR results showed that the MCM8 expression was reduced in HCCLM3 and Hep3B cells transfected with sh-EBP2. Data were shown as mean ± SD (*n* ≥ 3). ** *P* < 0.01, ns not significant.

To elucidate the molecular mechanism by which EBP2 regulates MCM8 expression, we first sought to identify potential transcription factors binding to the MCM8 promoter region. Using the UCSC Genome Browser, the 2 kb upstream sequence from the *MCM8* transcriptional start site was extracted and designated as the putative promoter region. In silico analysis using AnimalTFDB3.0 predicted several candidate transcription factors with potential binding affinity to this regulatory region. To assess the potential interaction between these transcription factors and EBP2, the STRING database was utilized for protein-protein interaction (PPI) network analysis. This analysis generated a hypothesis that EBP2 may regulate MCM8 expression via transcriptional co-factors, including STAT1, CENPA, YY1, MYC, E2F1, and TAF1 (Fig. 4A). Subsequently, the expression profiles of candidate transcription factors were analyzed following EBP2 knockdown. qRT-PCR showed significant changes in CENPA mRNA expression compared to other candidate transcription factors in HCCLM3 and Hep3B cells transfected with sh-EBP2 (Fig. 4B). WB analysis further validated these genes, demonstrating that EBP2 knockdown markedly downregulated CENPA protein expression (Fig. 4C). ChIP assays were conducted to determine whether CENPA binds to the MCM8 promoter and whether EBP2 modulates this binding interaction. The results demonstrated a significant increase in CENPA occupancy at the MCM8 promoter in EBP2-overexpressing cells compared to control cells, indicating that EBP2 promotes the recruitment of CENPA to the MCM8 promoter region (Fig. 4D). A co-IP assay was then conducted to investigate the physical interaction between EBP2 and CENPA. The results confirmed a direct protein-protein interaction between EBP2 and CENPA, supporting the hypothesis that EBP2 facilitates CENPA recruitment to the MCM8 promoter, thereby enhancing MCM8 transcription activation (Fig. 4E). To further validate the role of CENPA in activating the MCM8 promoter, a dual-luciferase reporter assay was performed. The MCM8 promoter region was cloned into a luciferase reporter vector, and the impact of CENPA overexpression or knockdown on promoter activity was evaluated. Results showed that CENPA overexpression significantly enhanced luciferase activity driven by the MCM8 promoter, whereas CENPA silencing attenuated this activity. Conversely, mutation of the predicted CENPA binding sites within the promoter region abrogated this effect in both overexpression and knockdown settings, confirming the specificity of CENPA-mediated activation of the MCM8 promoter (Fig. 4F, G). To further validate the regulatory interaction between CENPA and MCM8, qRT-PCR and WB analysis were conducted. The results demonstrated that overexpression of CENPA significantly upregulated the mRNA and protein levels of MCM8 in HCCLM3 cells, whereas knockdown of CENPA led to a reciprocal decrease in MCM8 expression, indicating that CENPA positively regulates MCM8 mRNA and protein expression (Fig. 4H, I). Finally, we examined MCM8 expression following EBP2 knockdown. Quantitative analysis revealed that MCM8 expression was significantly downregulated in HCCLM3 and Hep3B cells transfected with sh-EBP2, whereas no change was observed in sh-Ctrl transfection cells (Fig. 4J). Previous work identified EBP2 as a novel c-MYC interactor that forms a positive-feedback loop to regulate nucleolar c-MYC activity, cell proliferation and tumorigenesis [23]. We confirmed this interaction experimentally; co-IP verified a direct protein-protein interaction between EBP2 and c-MYC (Fig. S4A). We next asked whether this complex modulates MCM8 or HMGB1 expression. Efficient knockdown and over-expression vectors for MYC were first validated by qPCR, demonstrating robust modulation of MYC mRNA in HCCLM3 and Hep3B cells (Fig. S4B). WB subsequently showed that siRNA-mediated depletion of MYC reduced endogenous c-MYC protein without altering MCM8 or HMGB1 levels; conversely, ectopic over-expression of MYC failed to affect either MCM8 or HMGB1 expression (Fig. S4C). Collectively, these data indicate that, although EBP2 binds c-MYC, this interaction does not regulate MCM8 or HMGB1 expression in HCCLM3 or Hep3B cells.

### Functional rescue experiments demonstrate the role of MCM8 in EBP2-mediated HCC progression

**Fig. 5:**
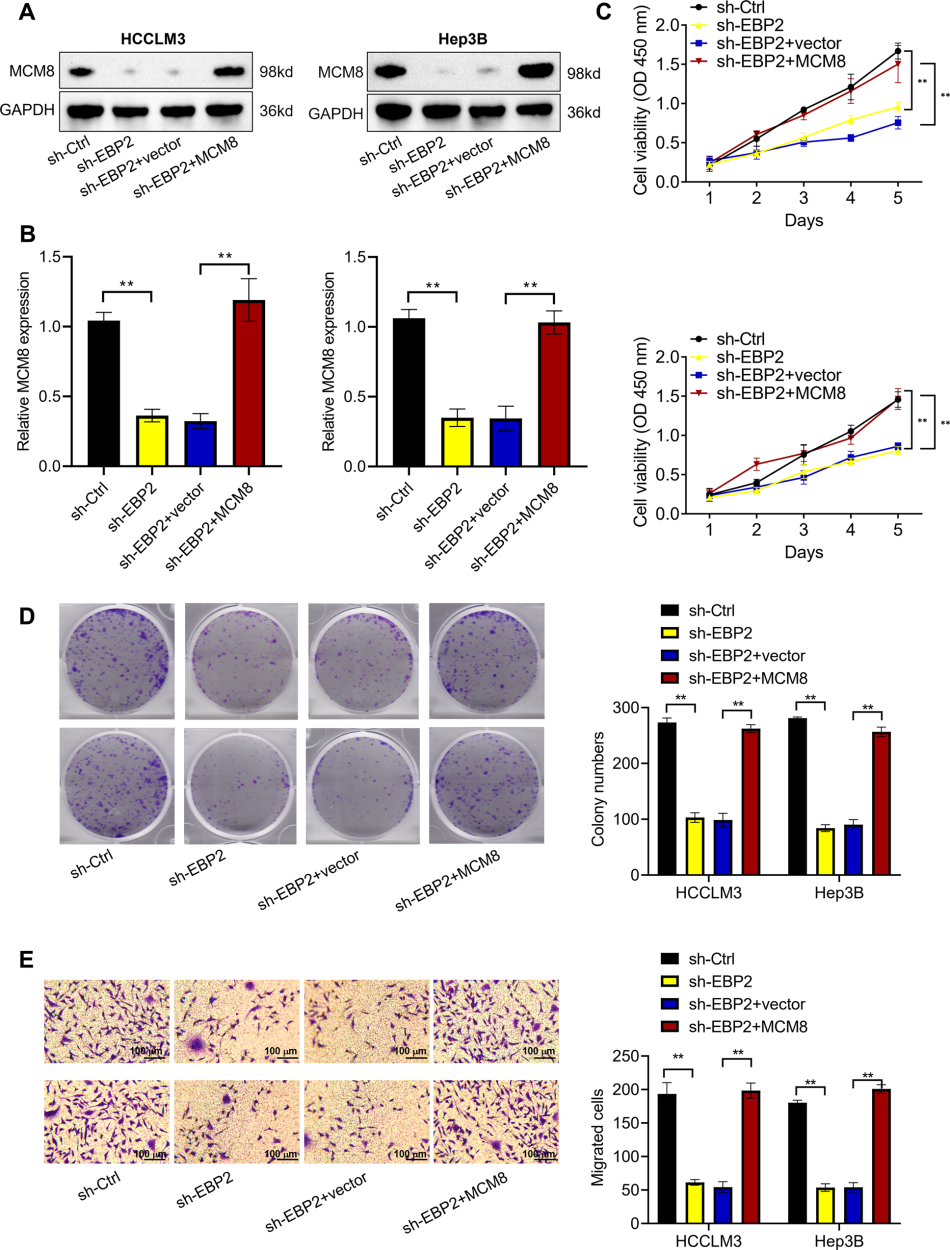
MCM8 overexpression rescues the inhibitory effects of EBP2 knockdown on HCC cell functions. **A**, **B** WB and qPCR analyses confirming MCM8 down-expression in HCCLM3 and Hep3B cells transduced with EBP2 knockdown, and subverted by co-transfected with MCM8-overexpressing lentiviral constructs. **C** CCK8 assays showing that EBP2 knockdown significantly suppresses cell proliferation, and this effect is reversed by co-transfection with MCM8-overexpressing lentiviral constructs. **D** Clonogenic assays demonstrating that EBP2 knockdown inhibits clonogenic ability and counteracts it by co-transfection with MCM8. **E** Transwell assays showing that EBP2 knockdown inhibits cell migration and MCM8 overexpression reverses the migration inhibition induced by EBP2 knockdown. Scale bar = 100 μm. Data were shown as mean ± SD (*n* ≥ 3). ** *P* < 0.01.

To evaluate the functional role of MCM8 in HCC progression, lentiviral constructs for MCM8 overexpression were generated and transduced into HCCLM3 and Hep3B cells. qPCR and WB analyses confirmed that EBP2 knockdown significantly downregulated MCM8 expression relative to the sh-Ctrl group, whereas co-transfection with an MCM8 expression vector restored MCM8 expression levels (Fig. 5A, B). The CCK8 assay results demonstrated that EBP2 downregulation significantly inhibited the proliferation of HCCLM3 and Hep3B cells. Notably, MCM8 overexpression markedly alleviated the proliferation inhibition caused by EBP2 knockdown, suggesting that MCM8 can restore the proliferative capacity suppressed by EBP2 depletion (Fig. 5C). Additionally, EBP2 knockdown significantly impaired the clonogenic potential of both HCCLM3 and Hep3B cells. Additionally, the reduction in colony formation induced by EBP2 knockdown was significantly rescued by MCM8 overexpression, further validating the role of MCM8 in counteracting the inhibitory effects of EBP2 depletion (Fig. 5D). Transwell assay revealed that EBP2 knockdown significantly impaired the migratory capacity of both HCCLM3 and Hep3B cells. Notably, MCM8 overexpression effectively attenuated the migration inhibition caused by EBP2 knockdown, suggesting that MCM8 plays a crucial role in HCC cell migration (Fig. 5E). These data demonstrate that EBP2 drives HCC cell proliferation and migration via transcriptional up-regulation of MCM8, establishing MCM8 as a requisite effector rather than a passive target of EBP2 oncogenic signaling.

### EBP2 promotes HCC progression by facilitating homology-directed DNA repair via MCM8/MCM9 complex assembly

**Fig. 6:**
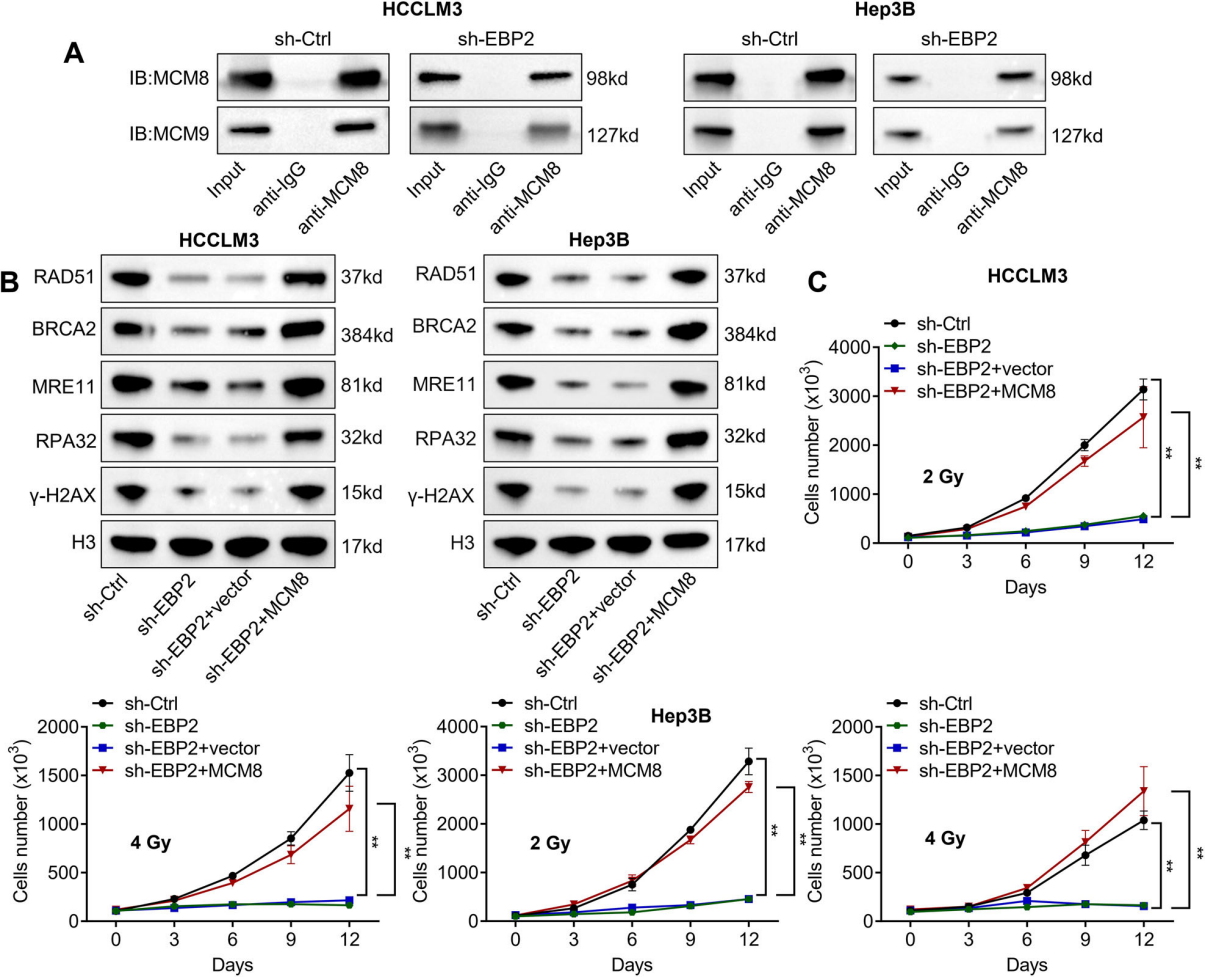
EBP2 promotes HCC progression by facilitating homology-directed DNA repair via MCM8/MCM9 complex assembly. **A** co-IP analyses confirmed the formation of a bona fide MCM8-MCM9 complex and demonstrated a marked reduction in complex abundance upon EBP2 depletion. **B** WB showing EBP2 depletion reduces RAD51, BRCA2, MRE11, RPA32 and γ-H2AX in HCCLM3 and Hep3B cells; co-expression of MCM8 rescues these defects. **C** Clonogenic assay showing EBP2 knockdown sensitises HCCLM3 and Hep3B cells to 2 Gy or 4 Gy IR, an effect rescued by MCM8 re-expression. Data were shown as mean ± SD (*n* ≥ 3). ** *P* < 0.01.

Next, we interrogated the contribution of DNA-damage repair (DDR) circuitry to HCC. Homologous recombination (HR) is indispensable for both germline DSB repair during mammalian gametogenesis and error-free resolution of exogenous- or endogenous-induced DSBs in somatic cells [24]. The minichromosome-maintenance proteins MCM8 and MCM9, recently identified HR factors, participate in DNA replication initiation, meiotic recombination, HR and mismatch repair [25, 26]. Genetic ablation of MCM8/9 in murine models engenders HR insufficiency, germ-cell failure and genomic instability, while their helicase complex formation has been implicated in tumor progression [27]. We therefore examined whether MCM8-mediated HCC pathobiology is MCM9-dependent. Co-IP confirmed that MCM8 and MCM9 assemble a stable complex whose abundance is diminished upon EBP2 knockdown (Fig. 6A). Next, we analysed chromatin recruitment of five HR proteins, including RAD51, a key homologous recombination factor [28]-BRCA2, MRE11, PALB2 and γ-H2AX. In HCCLM3 and Hep3B cells, EBP2 knockdown markedly attenuated RAD51 chromatin loading; analogous reductions were observed for the remaining four factors. Co-expression of MCM8, however, substantially restored recruitment of all five HR proteins (Fig. 6B). These observations indicate that, at the molecular level, EBP2 depletion compromises intracellular homologous recombination proficiency, whereas MCM8 overexpression ameliorates this defect. Ionising radiation (IR)-induced DNA double-strand breaks are the most lethal form of DNA damage and trigger a cascade of cellular DNA-damage responses that facilitate recovery from radiation injury [29]. Accordingly, we exposed HCCLM3 and Hep3B cells to IR at 2 Gy or 4 Gy and assessed clonogenic survival. EBP2 knockdown markedly reduced radio-resistance in both cell lines, whereas MCM8 co-expression substantially restored their ability to withstand IR exposure (Fig. 6C). Collectively, these data establish that EBP2 promotes HCC progression by licensing the MCM8/MCM9 complex to orchestrate HR-dependent DNA repair.

### EBP2 modulates CENPA/YY1 to enhance transcriptional control of HMGB1 and drive HCC proliferation

**Fig. 7:**
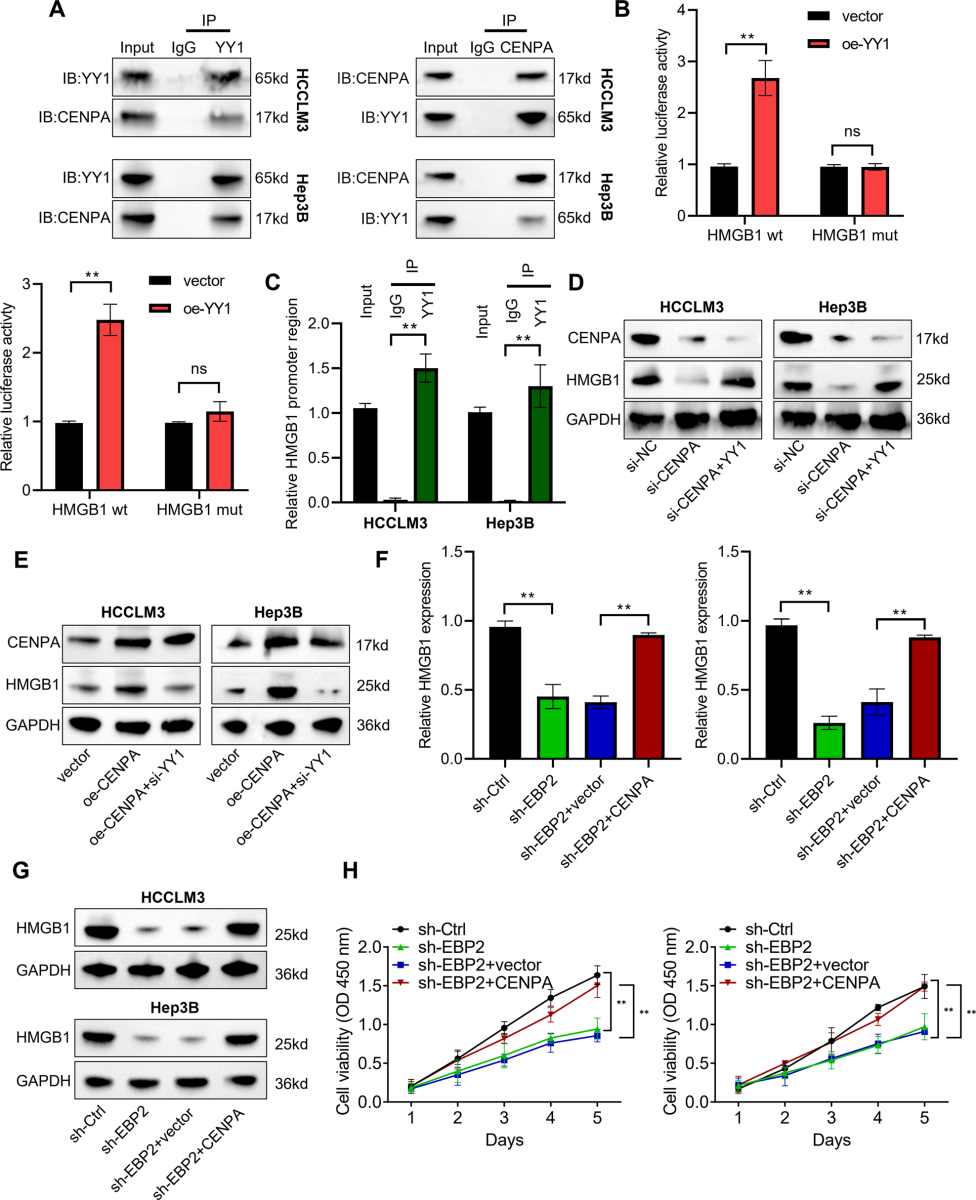
EBP2 modulates CENPA/YY1 to enhance transcriptional control of HMGB1 and drive HCC proliferation. **A** co-IP confirms a direct YY1-CENPA interaction in HCC cells. **B** Dual-luciferase assays reveal that YY1 overexpression activates the HMGB1 promoter and mutation of the YY1-binding sites abolishes this activation. **C** ChIP-qPCR demonstrates that YY1 occupies the HMGB1 promoter, confirming its role as a direct transcriptional regulator. **D** WB indicates that CENPA knockdown reduces HMGB1, and co-transfection with YY1 restores the protein level. **E** WB further shows that CENPA overexpression up-regulates HMGB1, whereas YY1 knockdown reverses this increment. **F**, **G** qPCR and WB jointly show that EBP2 knockdown down-regulates HMGB1, and co-transfection with CENPA rescues the expression. **H** CCK-8 assays reveal that EBP2 knockdown suppresses HCCLM3 and Hep3B proliferation, an effect partially alleviated by CENPA overexpression. Data were shown as mean ± SD (*n* ≥ 3). ** *P* < 0.01.

Given our demonstration that EBP2 governs CENPA, together with prior evidence implicating HMGB1 in HCC progression, we hypothesised that EBP2 might modulate HMGB1 expression through CENPA, thereby promoting HCC. However, interrogation of transcription-factor databases indicated that CENPA does not occupy the HMGB1 promoter, implying that CENPA cannot directly control HMGB1 transcription as it does for MCM8. Nevertheless, independent work has revealed that CENPA interacts with YY1 to form a CENPA/YY1 complex that enhances CCND1 and NRP2 transcription and drives hepatocellular proliferation [30]. Furthermore, YY1 itself binds the HMGB1 promoter and regulates its transcription [31]. These findings prompted us to posit that aberrant HMGB1 expression in HCC is governed by the CENPA/YY1 transcriptional module. To formally address this hypothesis, we first performed co-IP assays to interrogate a direct physical interaction between YY1 and CENPA. These experiments revealed robust and specific assembly of a YY1–CENPA complex in human HCC cell lines (Fig. 7A). To delineate the functional consequence of this interaction in HMGB1 transcriptional control, we next asked whether YY1 engages the HMGB1 promoter. A 2-kb fragment of the HMGB1 promoter was cloned into a pGL3-basic luciferase reporter, and luciferase activity was quantified after ectopic YY1 expression. YY1 overexpression markedly augmented HMGB1 promoter-driven luciferase output, whereas site-directed mutagenesis of the predicted YY1 consensus motifs abrogated this activation, thereby confirming the specificity of YY1-mediated transcriptional activation of the HMGB1 promoter (Fig. 7B). Subsequently, ChIP followed by quantitative PCR (ChIP-qPCR) using an anti-YY1 antibody demonstrated marked and specific enrichment at the HMGB1 promoter relative to isotype control IgG, establishing YY1 as a direct transcriptional regulator of HMGB1 (Fig. 7C). WB analyses confirmed that CENPA knockdown markedly attenuated both CENPA and HMGB1 protein levels relative to the si-NC group, whereas co-transfection of a YY1-overexpression plasmid fully restored HMGB1 expression without altering CENPA abundance (Fig. 7D), indicating that YY1 functions downstream of CENPA. Conversely, enforced CENPA expression significantly elevated HMGB1 levels, and this induction was reversed upon YY1 depletion. Collectively, these data demonstrate that the CENPA-YY1 transcriptional axis cooperatively drives HMGB1 expression in HCC (Fig. 7E). Next, we interrogated the upstream regulatory circuitry. Both qPCR and immunoblot analyses revealed that EBP2 depletion significantly reduced HMGB1 transcript and protein levels when compared with the sh-Ctrl group, whereas co-delivery of a CENPA-overexpression construct fully rescued HMGB1 expression (Fig. 7F, G). Concordantly, CCK-8 assays demonstrated that EBP2 knockdown markedly attenuated the proliferation of HCCLM3 and Hep3B cells; importantly, ectopic CENPA expression substantially alleviated this growth arrest, indicating that CENPA-mediated HMGB1 induction is sufficient to counteract the anti-proliferative effect triggered by EBP2 loss (Fig. 7H). Collectively, these findings establish that EBP2 governs HCC proliferation by transcriptionally amplifying the CENPA-YY1-HMGB1 axis.

## Discussion

EBP2 is best characterized as a host cell protein that interacts with EBNA1, the viral latent protein EBNA1 is expressed in all EBV-associated tumors. EBNA1 is unique in that it represents the only latent protein expressed in certain EBV-driven malignancies, underscoring its critical role in viral genome maintenance, replication, and segregation. As a multifunctional regulator of viral episomal persistence, EBNA1 has emerged as a validated therapeutic target for EBV-associated cancers [32–35]. For instance, Soldan et al. reported that the EBNA1 inhibitor VK-1727 significantly arrested cell cycle progression and suppressed cell proliferation in vitro, while dose-dependently inhibiting the growth of EBV-associated gastric cancer xenografts in vivo. Notably, this inhibitor exhibited no antitumor activity against EBV-negative gastric cancer xenografts [33]. Conversely, EBP2 remains understudied in both EBV-associated and non-EBV-associated malignancies. Previous investigations have primarily linked EBP2’s involvement in malignancy to its interaction with the tumor suppressor FBXW7 (F-box and WD repeat domain-containing 7) [36–38]. Studies have demonstrated that EBP2 functions as a substrate for FBXW7, serving as a critical nucleolar factor that directly mediates the nucleolar targeting of FBXW7. Concurrently, EBP2 acts as a ubiquitination substrate for the E3 ubiquitin ligase complex (Skp1-Cullin1-F-box, SCF) formed by FBXW7, undergoing SCF-mediated ubiquitin-dependent degradation. Additionally, Liao et al. reported that EBP2 reduces c-Myc degradation and promotes its nucleolar localization in an FBXW7-independent manner, thereby inducing c-Myc-associated rRNA synthesis and facilitating cell proliferation [23]. Recent investigations have revealed that EBP2 deficiency triggers p53 activation via the Akt-mTORC1 signaling axis, leading to reduced proliferative capacity in NPM-ALK (nucleophosmin-anaplastic lymphoma kinase) fusion protein-positive anaplastic large cell lymphoma cells [39]. Building upon these varied functions and previous findings, the present comprehensive study explores for the first time the specific functional and mechanistic role of EBP2 in HCC, primarily a non-EBV-associated malignancy. IHC analysis demonstrated significantly elevated EBP2 expression in HCC tissues compared to normal hepatic tissues, with a clear correlation between EBP2 levels and advancing tumor stages. The tumor-promoting role of EBP2, as evidenced by clinical sample analyses, was further validated in both in vitro and in vivo models. Silencing of EBP2 in HCC cells significantly inhibited cell proliferation, induced apoptosis, and reduced cell migration capacity, while correspondingly suppressing the growth of subcutaneous xenografts in vivo.

MCM8, a conserved member of the minichromosome maintenance (MCM) family, has emerged as a focal point in recent investigations, particularly in studies examining its functional interplay with MCM9 [25, 40–43]. Trakselis et al. demonstrated that MCM8 functions as a DNA helicase during the elongation phase of DNA replication, facilitating the recruitment of RPA34 and augmenting DNA polymerase activity at replication [44]. Numerous studies have established that the MCM8-MCM9 complex is critical for DNA repair and homologous recombination. Nielsen et al. reported that biallelic MCM9 variants are associated with polyposis, gastric cancer, and early-onset CRC, while both biallelic MCM8 and MCM9 variants are linked to hypogonadism and the early development of germ cell tumors [45]. Moreover, genetic ablation of MCM9 or knockdown of MCM8 has been shown to selectively render cancer cells sensitive to cisplatin and Olaparib. Mechanistic studies indicate that MCM8 depletion enhances cellular sensitivity to these agents by exacerbating oncogene-induced replication stress [46]. Given MCM8’s well-documented oncogenic activity and its significant enrichment and clinical relevance observed in our EBP2-driven transcriptomic analyses, we selected it as a downstream effector of EBP2. Subsequent validations revealed that MCM8 is markedly upregulated in HCC tissues and strongly associates with unfavorable patient prognosis. Crucially, we observed decreased MCM8 expression in EBP2 knockdown cells and demonstrated that MCM8 overexpression can attenuate the inhibitory effects of EBP2 depletion on HCC progression. To elucidate how MCM8 promotes HCC progression, we conducted an in-depth mechanistic investigation that corroborated previous functional reports: MCM8 forms a stoichiometric complex with MCM9 to orchestrate DNA replication and damage repair, and this MCM8/MCM9 complex is markedly diminished upon EBP2 depletion. These data indicate that EBP2 licenses HR-mediated DNA repair via stabilization of the MCM8/MCM9 holocomplex, thereby fostering HCC evolution. Mechanistically, although EBP2 has been shown to interact with c-MYC, co-IP assays confirmed this physical association, yet functional analyses revealed that the EBP2-c-MYC interface does not impinge upon MCM8 transcriptional output. Collectively, these observations suggest that the EBP2-c-MYC interaction does not operate through a canonical transcriptional axis to mediate the pro-tumorigenic actions of MCM8 in HCC. In the present study, we provide the first demonstration that EBP2 transcriptionally up-regulates MCM8 through the centromeric histone H3 variant and transcriptional co-factor CENPA. As the foundational scaffold for outer kinetochore assembly [47]. CENPA has more recently been implicated in transcriptional activation and oncogenesis [48]. ChIP analyses revealed that CENPA directly occupies the MCM8 promoter and that EBP2 significantly enhances CENPA recruitment to this locus, a finding that aligns with recent genome-wide occupancy studies [49].

The architectural chromatin protein HMGB1 undergoes stimulus-evoked nuclear-to-cytoplasmic translocation and subsequent extracellular release, thereby functioning as a bona fide damage-associated molecular pattern (DAMP) [50]. Aberrant HMGB1 expression is a pathogenic hallmark of diverse inflammatory and neoplastic diseases [51]. In HCC, heightened HMGB1 bioavailability amplifies tumour-promoting inflammation, neo-angiogenesis, and resistance to sorafenib or cisplatin, effects that are predominantly transduced via Toll-like receptor 4 (TLR4)- or receptor for advanced glycation end-product (RAGE)-dependent signaling cascades [52, 53]. Collectively, the well-documented oncogenic properties of HMGB1, together with our unbiased data-mining and rigorous clinical correlation analyses in the present study, constituted the decisive rationale for designating HMGB1 as a bona fide downstream effector of EBP2. As an ancillary observation, we noted that HMGB1, similar to MCM8, is markedly up-regulated in HCC specimens and portends dismal clinical outcome. Mechanistically, EBP2 depletion precipitated a pronounced reduction in HMGB1 abundance, an effect partially rescued by concomitant enforced expression of either CENPA or YY1, underscoring that EBP2 governs HMGB1 transcription via the CENPA/YY1 co-activator complex. Mirroring the scenario observed for MCM8, the physical interaction between EBP2 and c-MYC did not modulate HMGB1 expression, indicating that this specific protein–protein interface is unlikely to operate through canonical transcriptional circuitry to mediate HMGB1-driven hepatocarcinogenesis.

However, this study has certain limitations, primarily the lack of clinical validation of the identified pathway. Therefore, future investigations should prioritize translating these findings to the clinical setting through rigorous validation in patient cohorts and exploration of therapeutic implications.

**Fig. 8:**
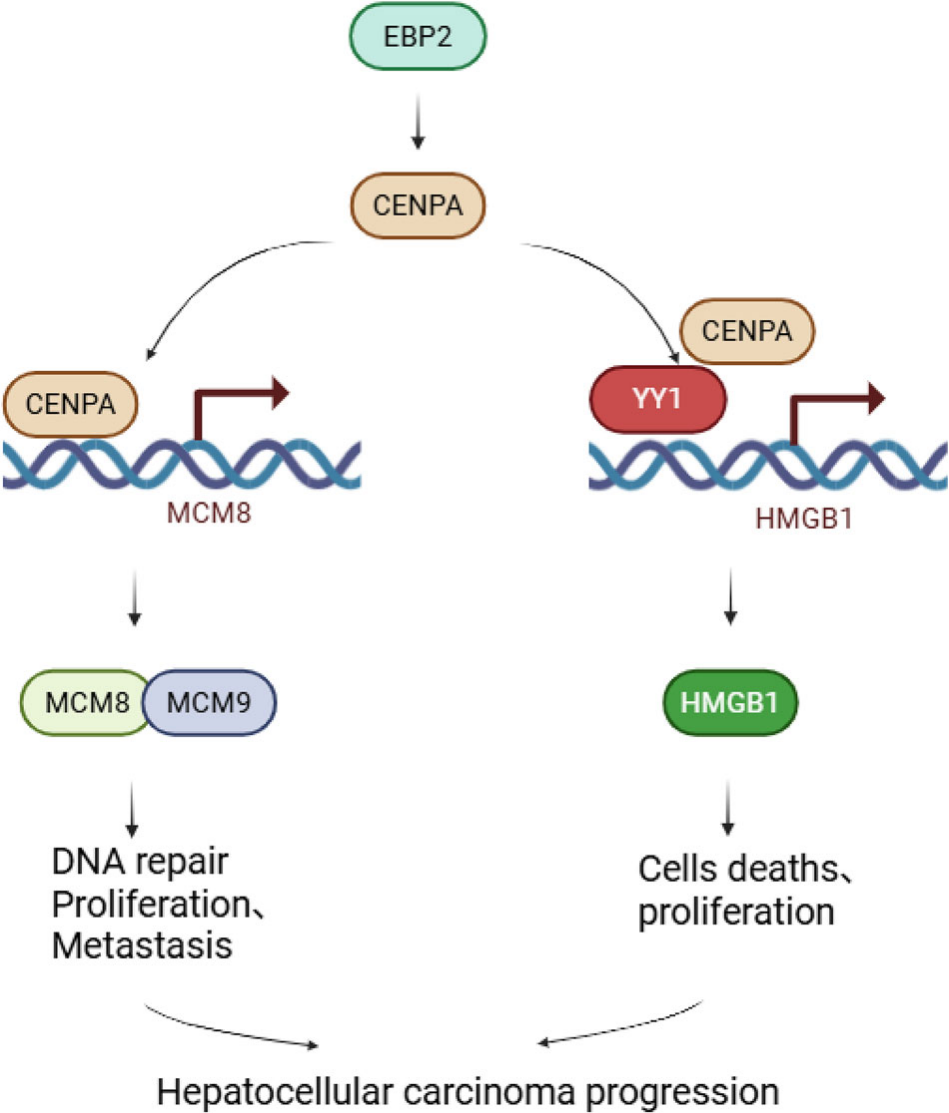
The hypothetical mechanistic model of EBP2-regulated MCM8 and HMGB1 axis in HCC progression. EBP2-CENPA drives MCM8 and HMGB1 transcription, stabilizing MCM8/9 for HR repair and activating CENPA/YY1-HMGB1 signaling in HCC.

In conclusion, this study demonstrates that EBP2 is significantly overexpressed in HCC tissues and is associated with poor patient prognosis. Functional analyses reveal that EBP2 knockdown in HCC cells suppresses cell proliferation, clonogenic potential, and migratory capacity while promoting apoptosis. In vivo studies further show that EBP2 depletion inhibits tumor growth in xenograft models. Mechanistically, EBP2 engages CENPA to transcriptionally up-regulate MCM8, thereby stabilizing the MCM8-MCM9 helicase complex and potentiating homologous recombination-executed DNA repair. Concomitantly, EBP2 orchestrates HMGB1 expression via the CENPA/YY1 co-activator complex (Fig. 8). These two parallel axes, namely, EBP2-CENPA-MCM8 and EBP2-CENPA/YY1-HMGB1, function in concert to drive HCC cell proliferation, migratory capacity, and survival. Collectively, these data establish EBP2 as both a robust potential prognostic biomarker and tractable therapeutic vulnerability in HCC.

## Supplementary information

Original Data

Supplementary Material

## Data Availability

The datasets used and/or analyzed during the current study are available from the corresponding author on reasonable request.
