# Supplementary material for: EBNA1BP2 (EBP2) promotes the progression of hepatocellular carcinoma through upregulating the expression of MCM8 and HMGB1
Source: Cell Death Dis. 2026 Apr 4;17(1):596. doi: 10.1038/s41419-026-08671-8 (PMC13303876; doi:10.1038/s41419-026-08671-8)
Supplement: Supplementary file 1 — Original Data [file 41419_2026_8671_MOESM1_ESM.pdf]

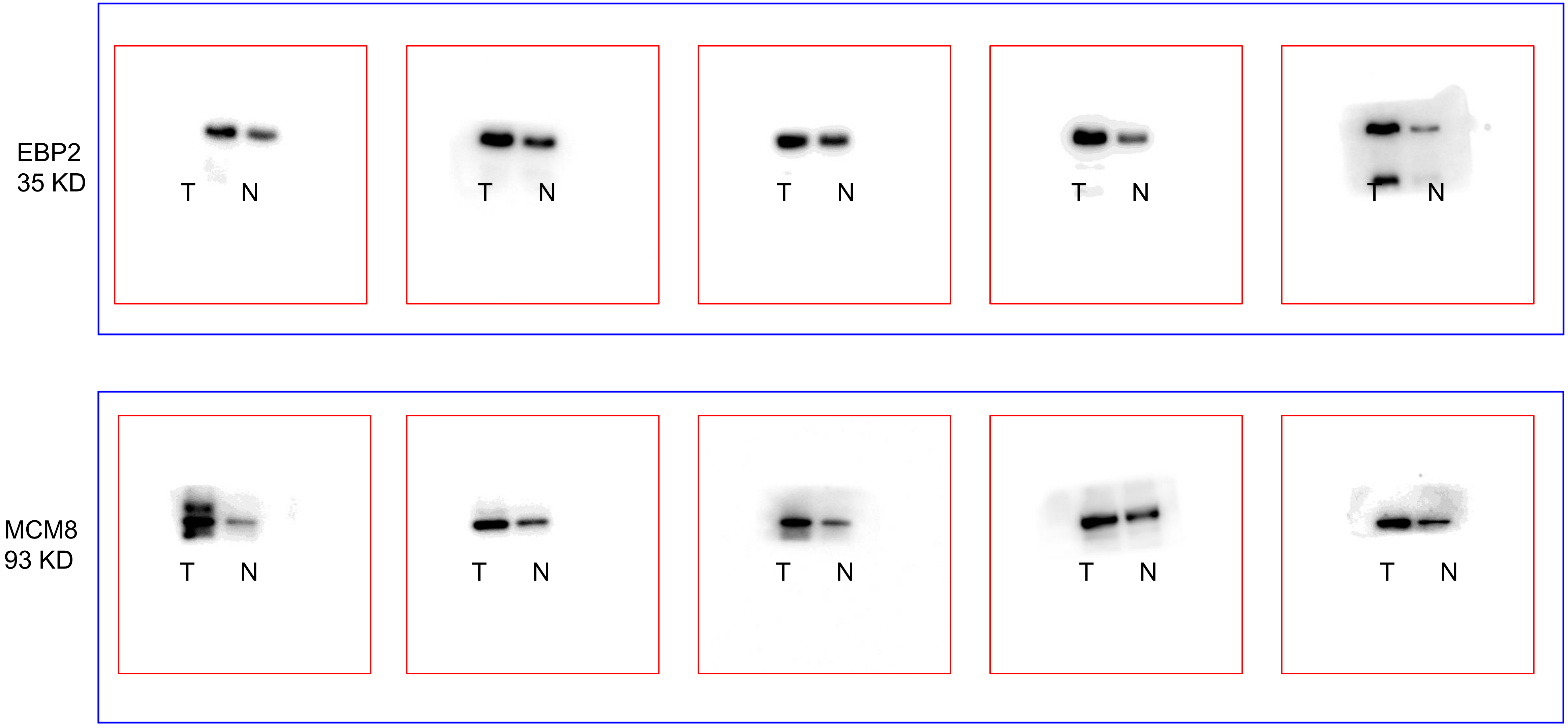

Raw data of WB for Figure 3H (EBP2 and MCM8)

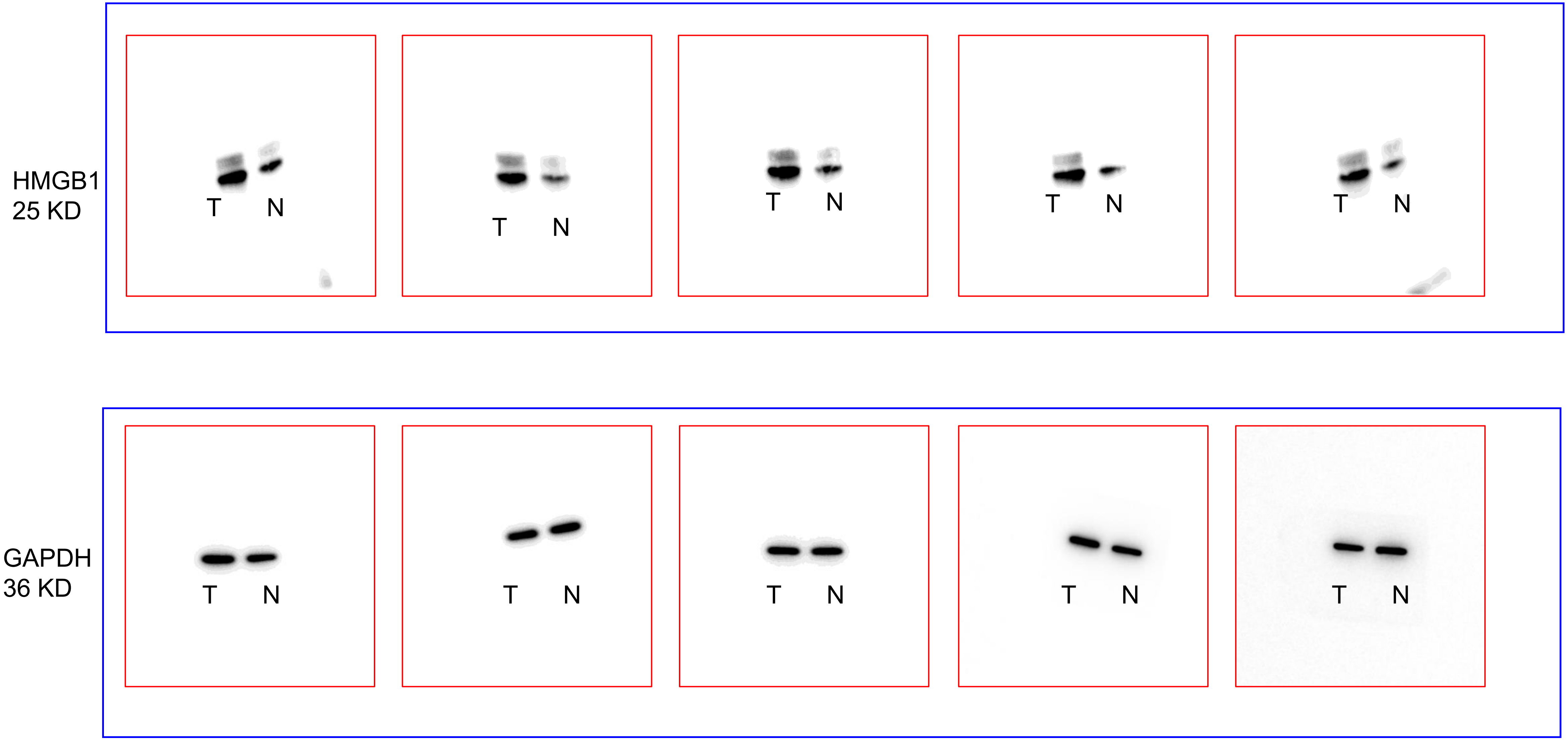

Raw data of WB for Figure 3H (EBP2 and MCM8)

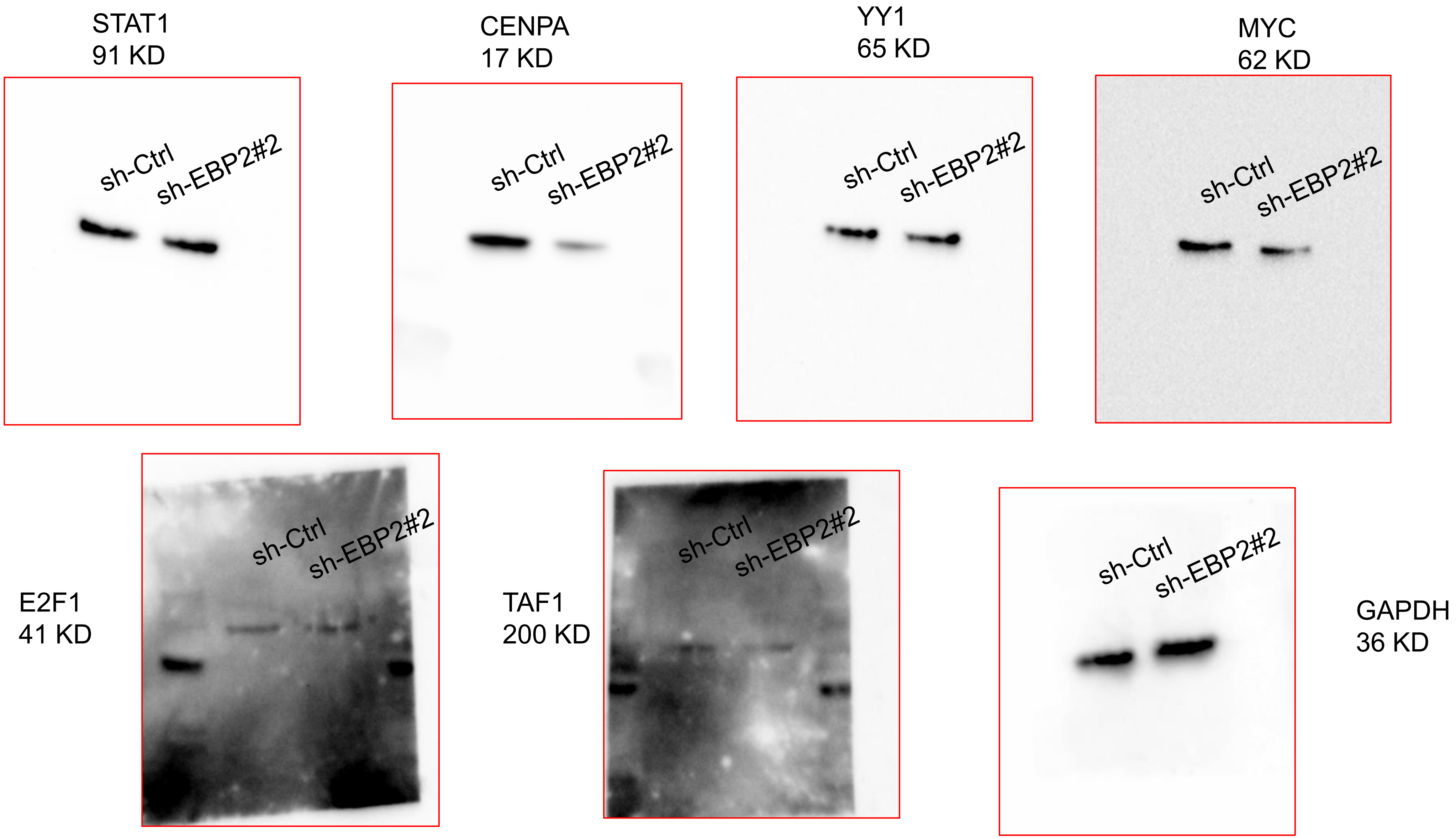

Raw data of WB for Figure 4C (upper panel in HCCLM3 cells)

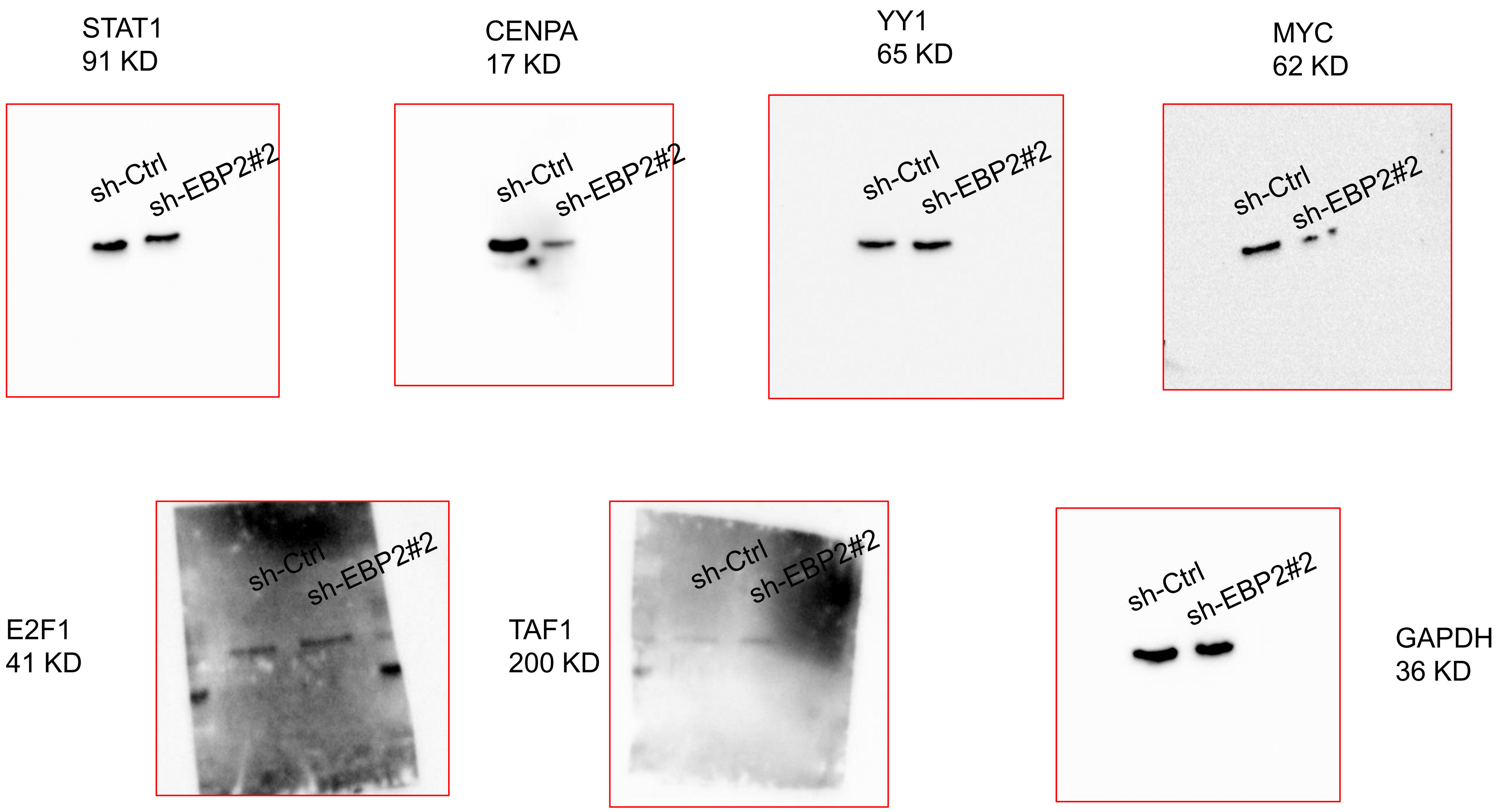

Raw data of WB for Figure 4C (upper panel in Hep3B cells)

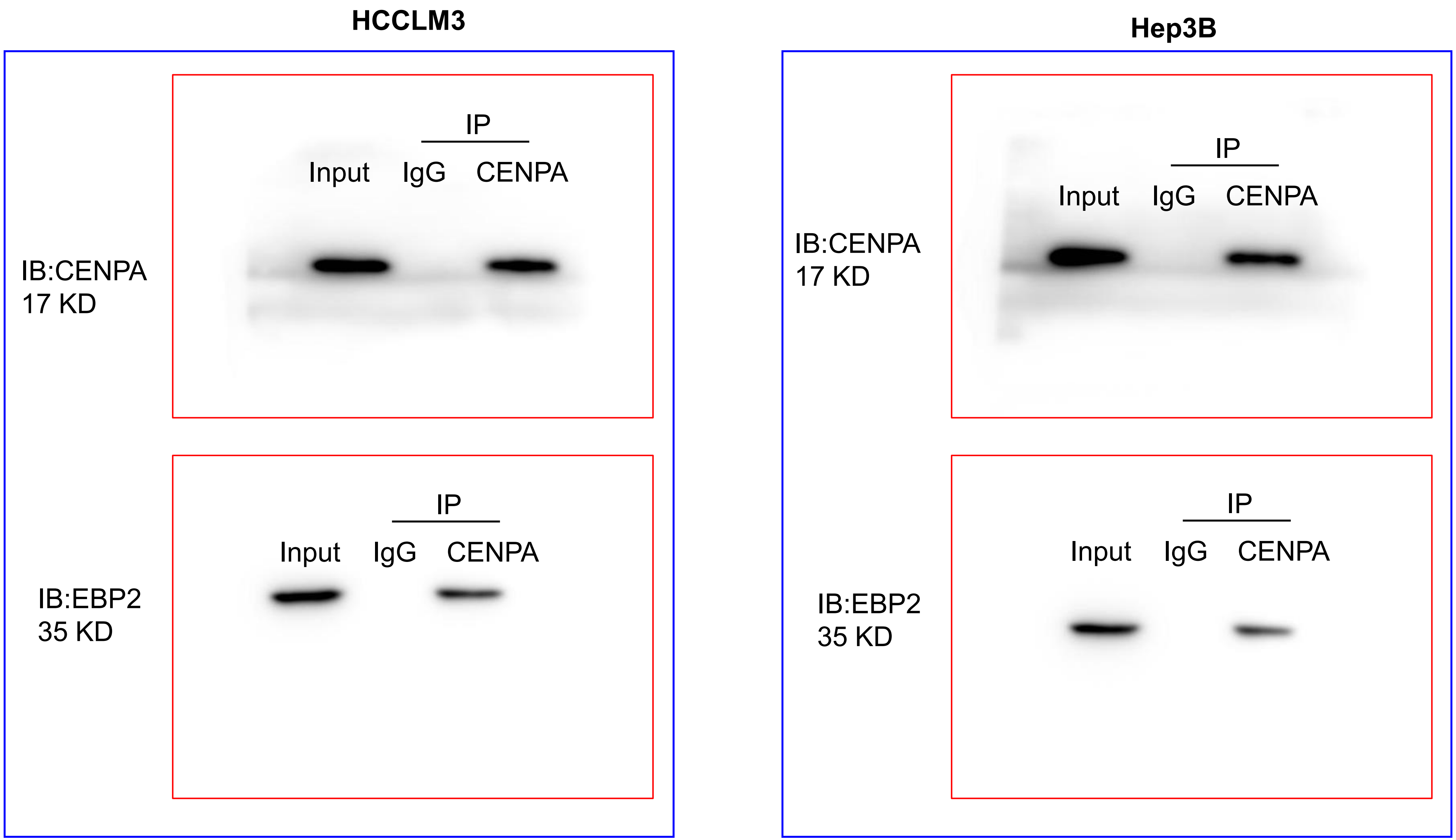

Raw data of WB for Figure 4E (upper panel in HCCLM3 and Hep3B cells)

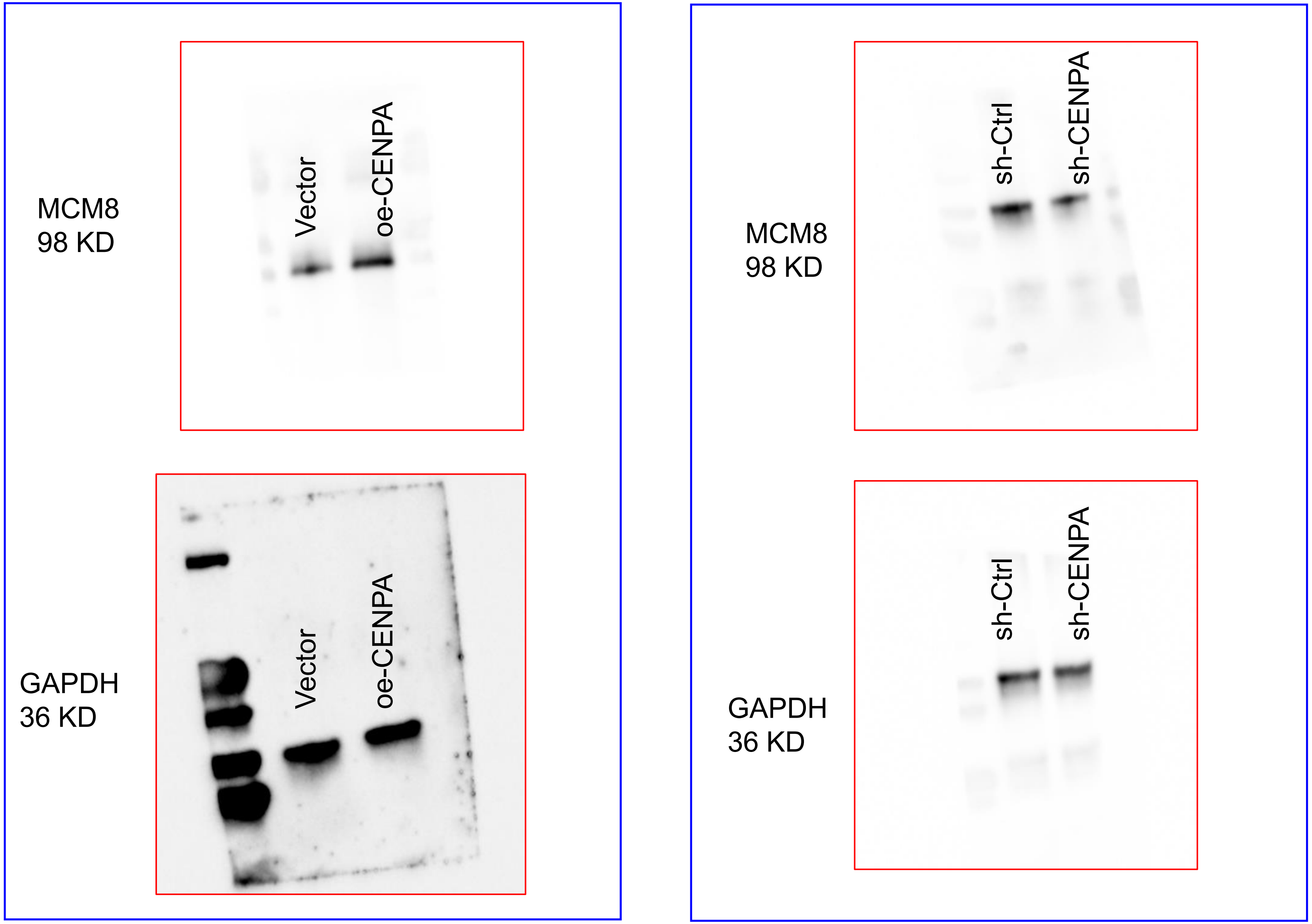

Raw data of WB for Figure 4I (upper panel in HCCLM3 cells)

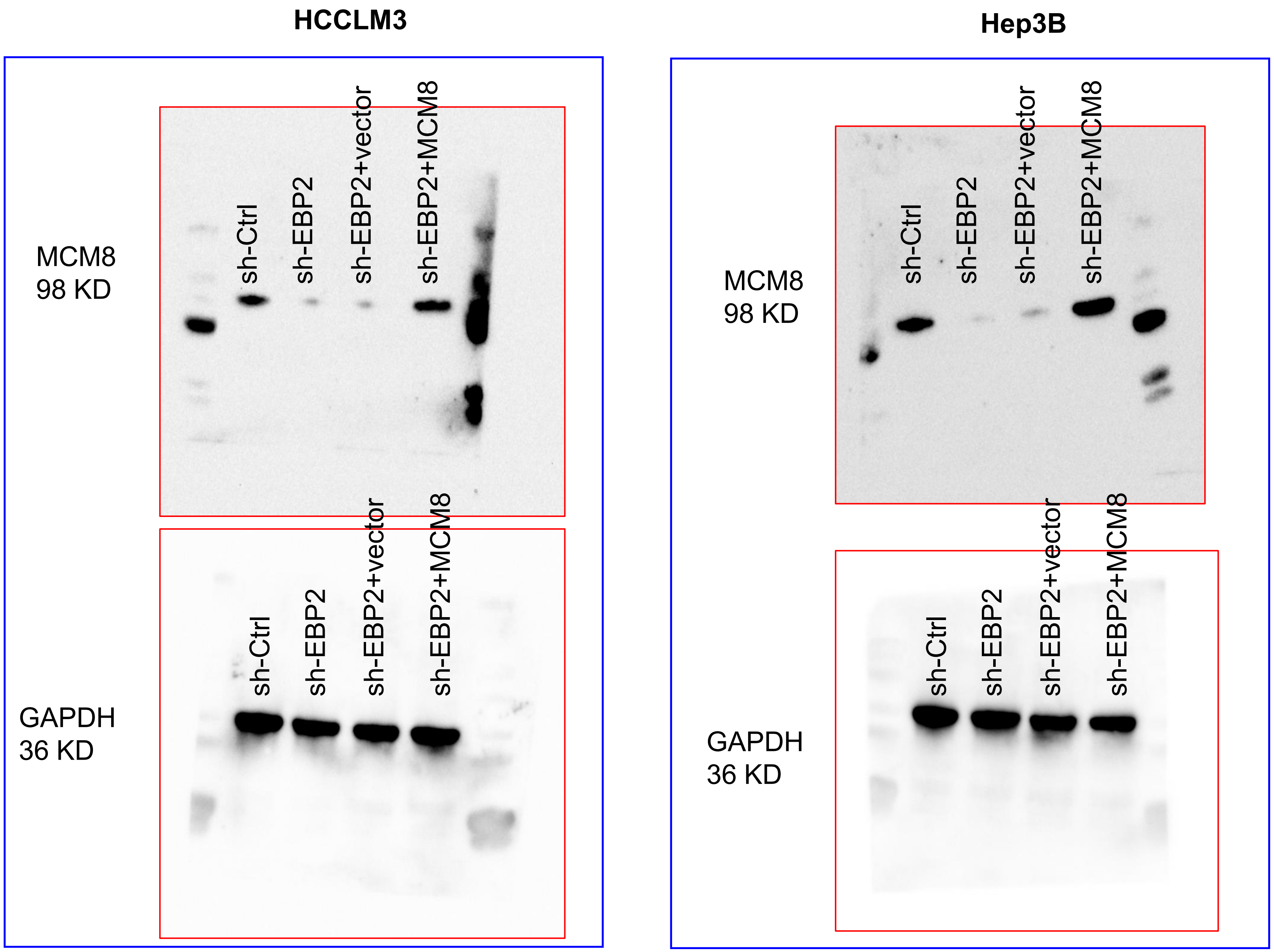

Raw data of WB for Figure 5A (upper panel in HCCLM3 and Hep3B cells)

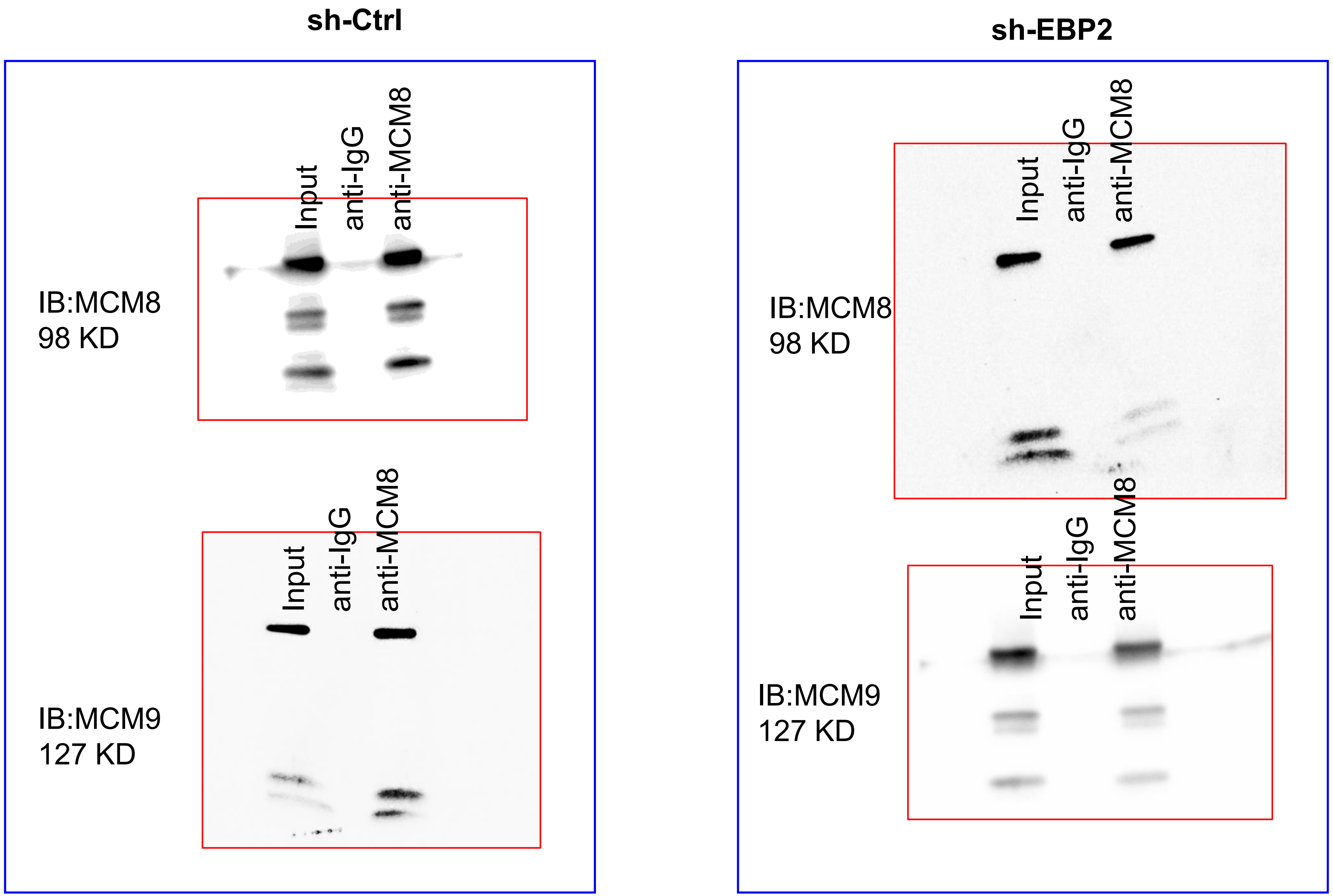

Raw data of WB for Figure 6A (upper panel in HCCLM3 cells)

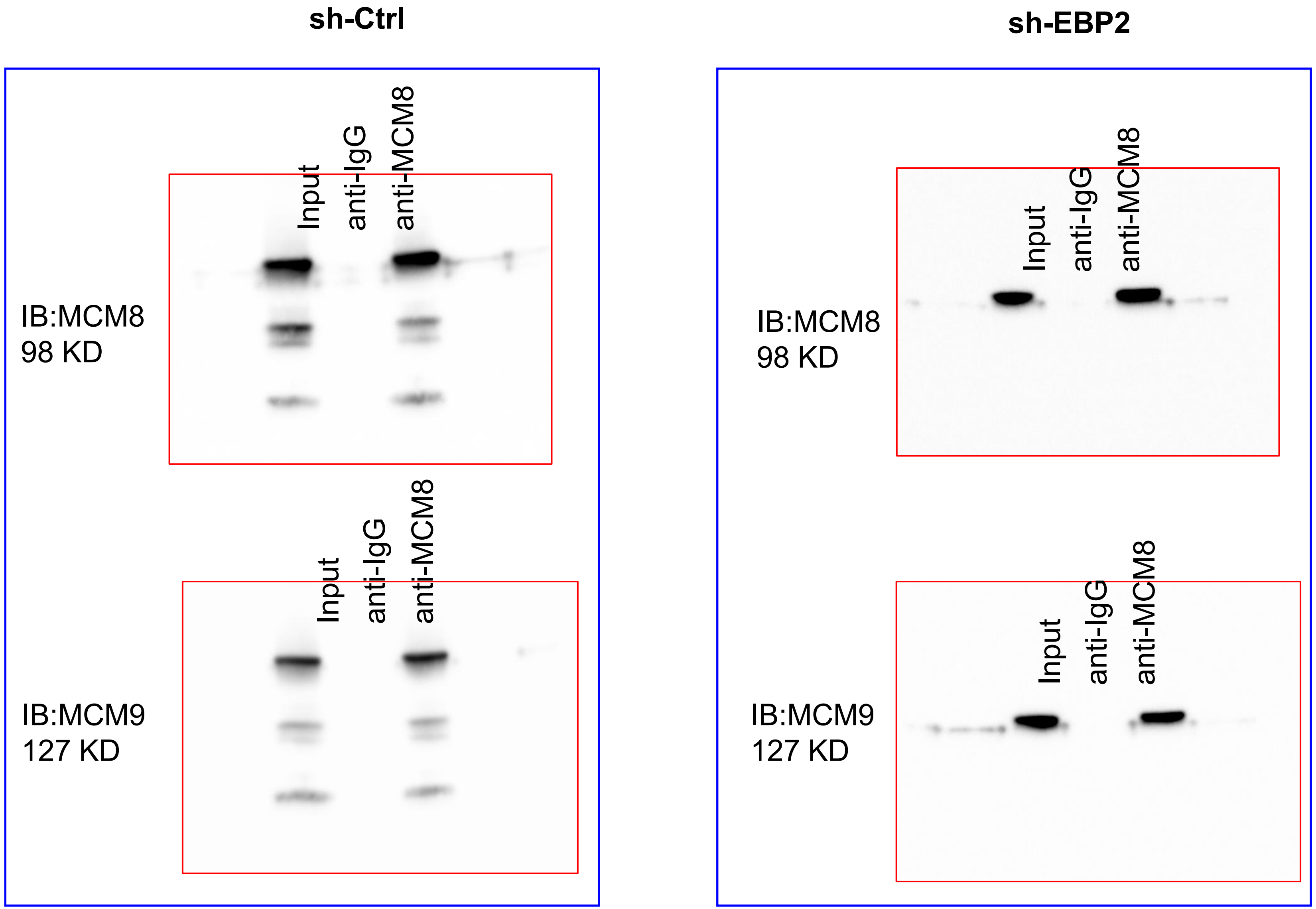

Raw data of WB for Figure 6A (upper panel in Hep3B cells)

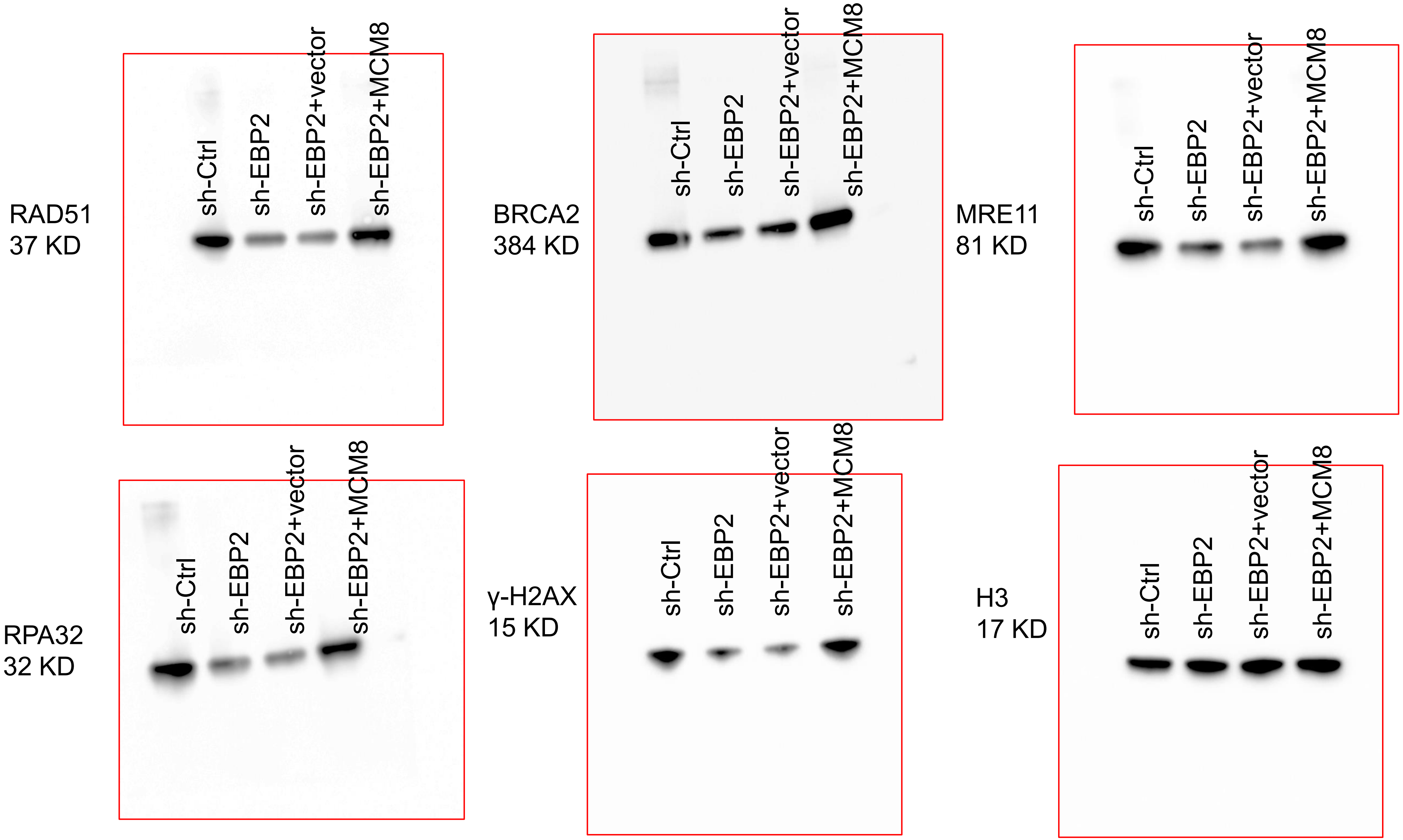

Raw data of WB for Figure 6B (upper panel in HCCLM3 cells)

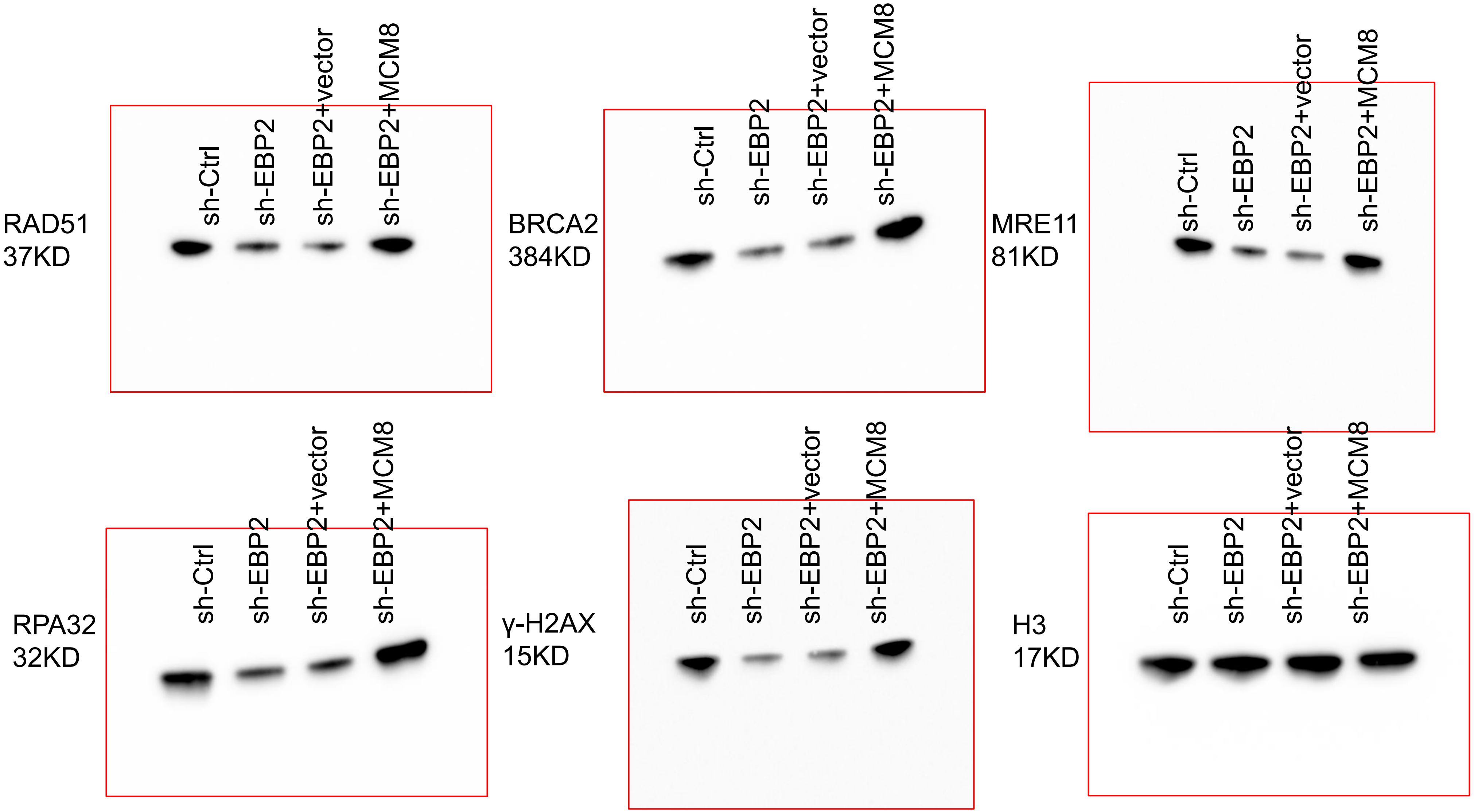

Raw data of WB for Figure 6B (upper panel in Hep3B cells)

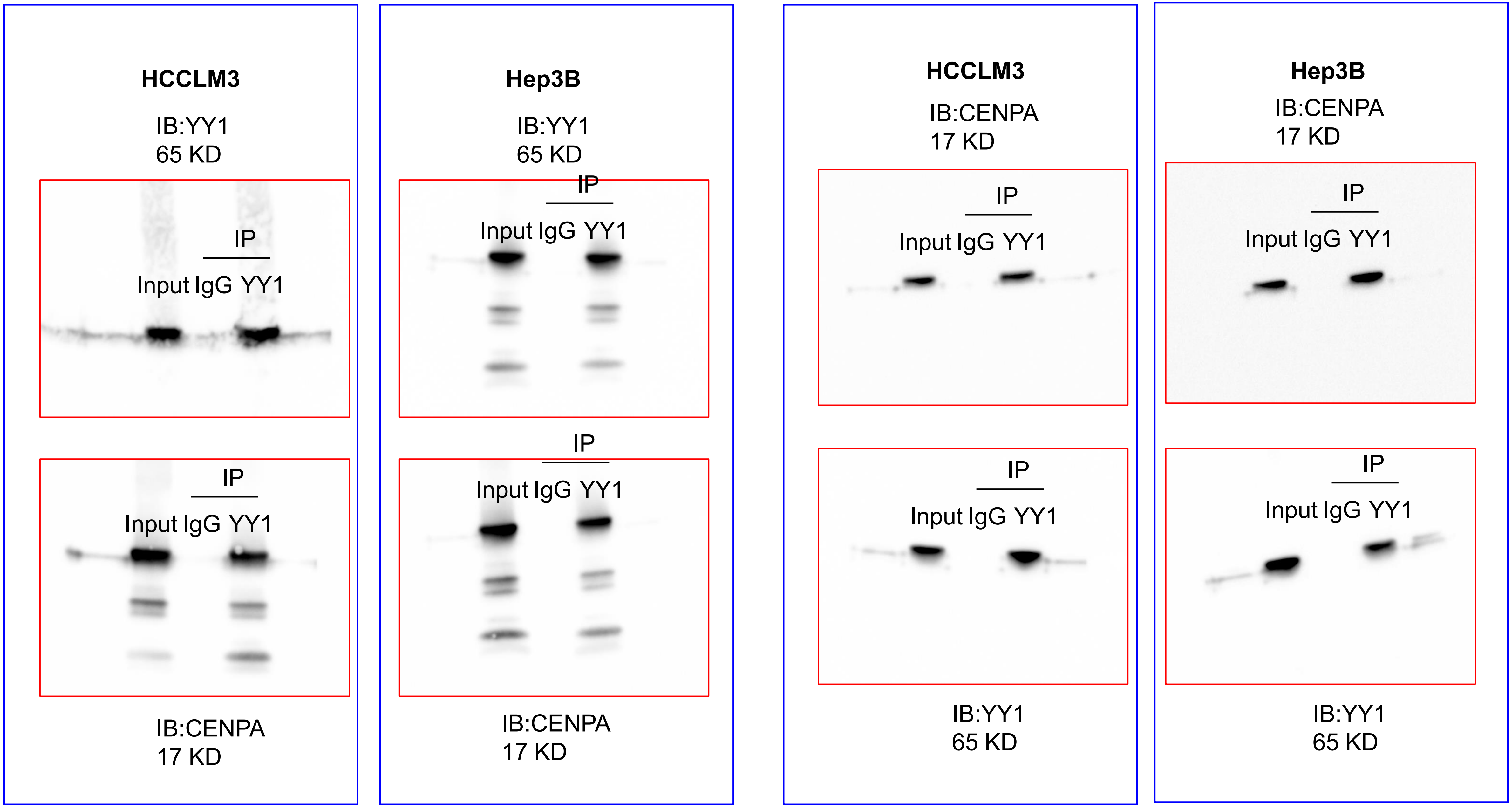

Raw data of WB for Figure 7A (upper panel in HCCLM3 and Hep3B cells)

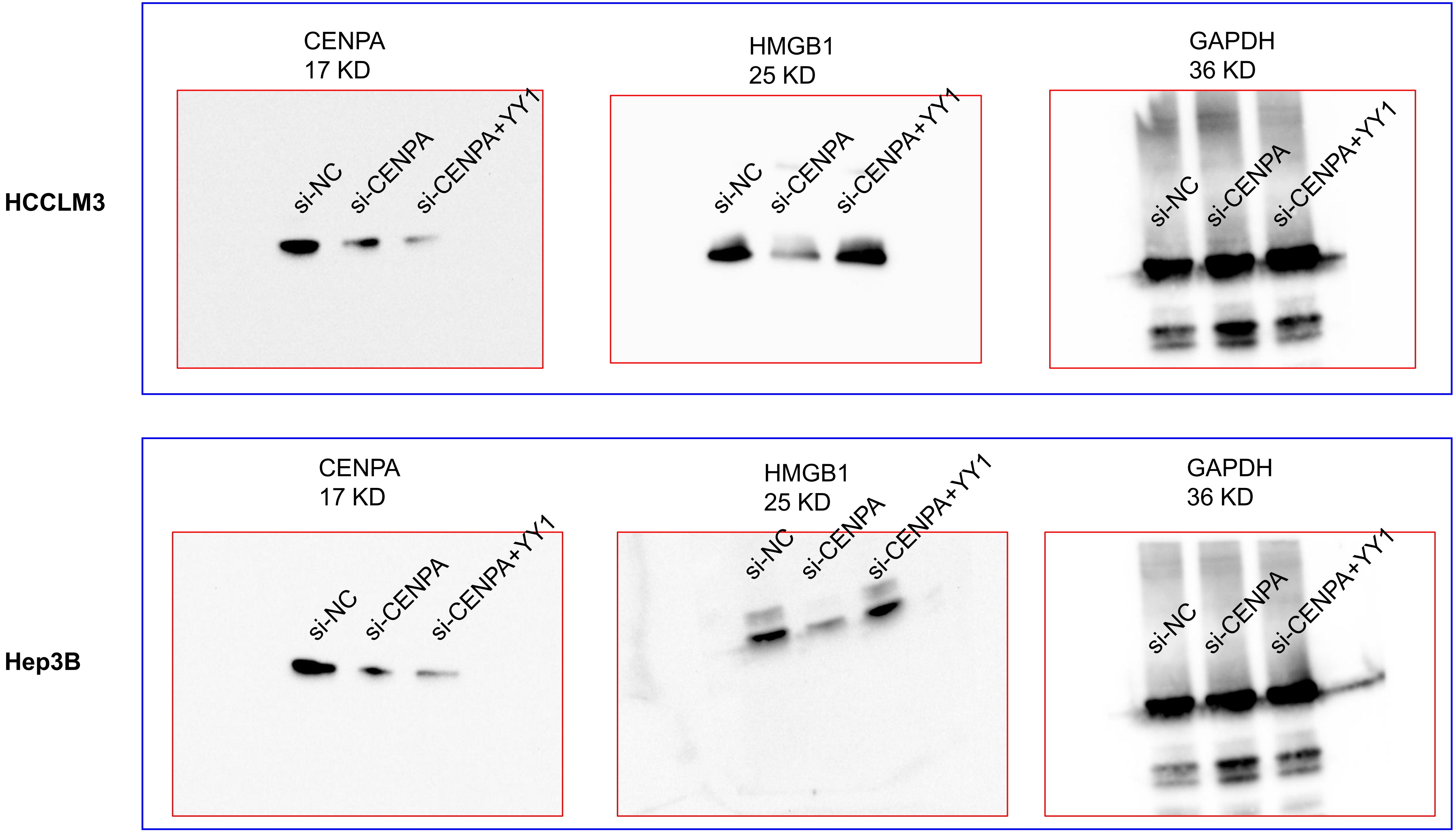

Raw data of WB for Figure 7D (upper panel in HCCLM3 and Hep3B cells)

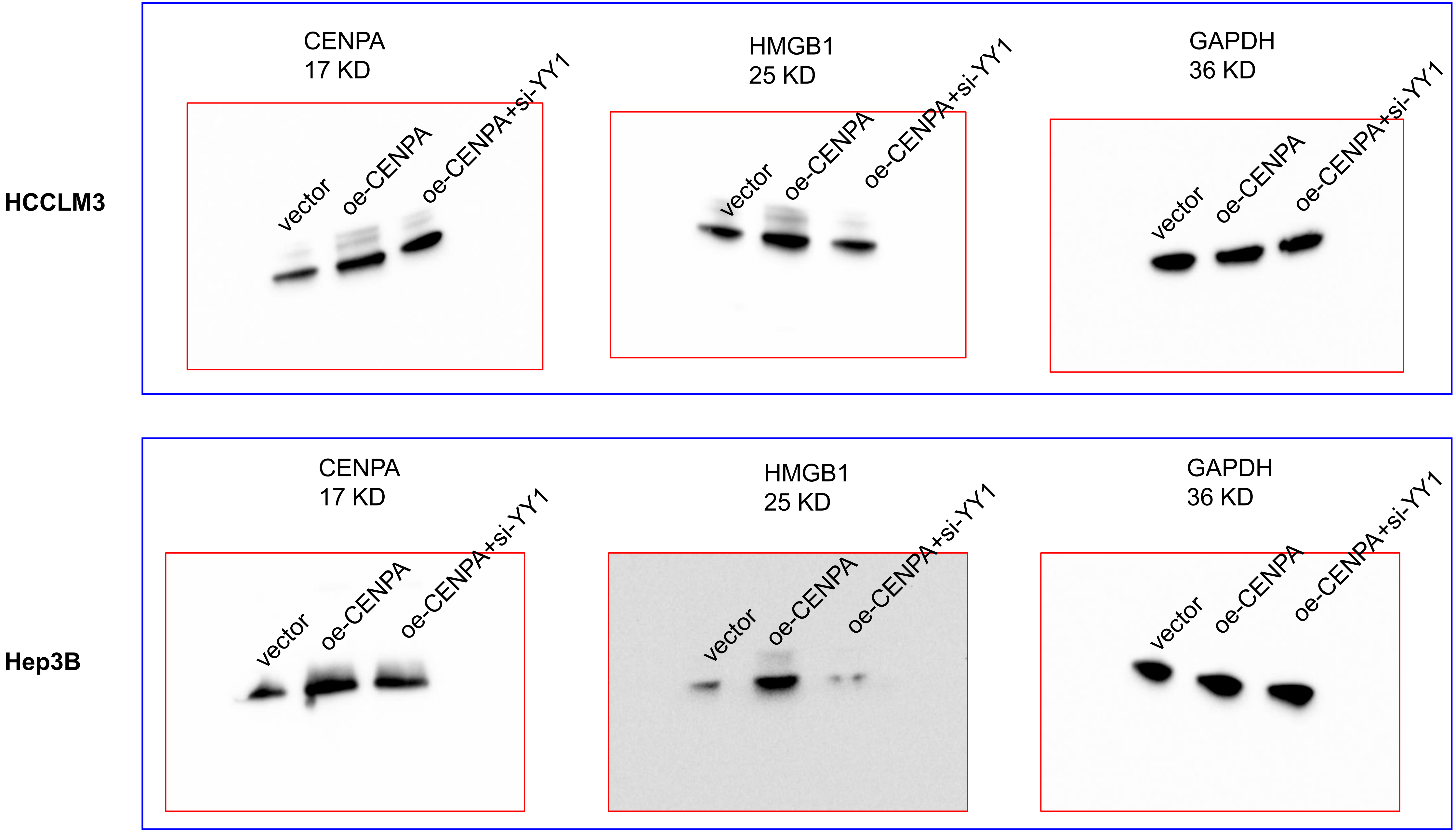

Raw data of WB for Figure 7E (upper panel in HCCLM3 and Hep3B cells)

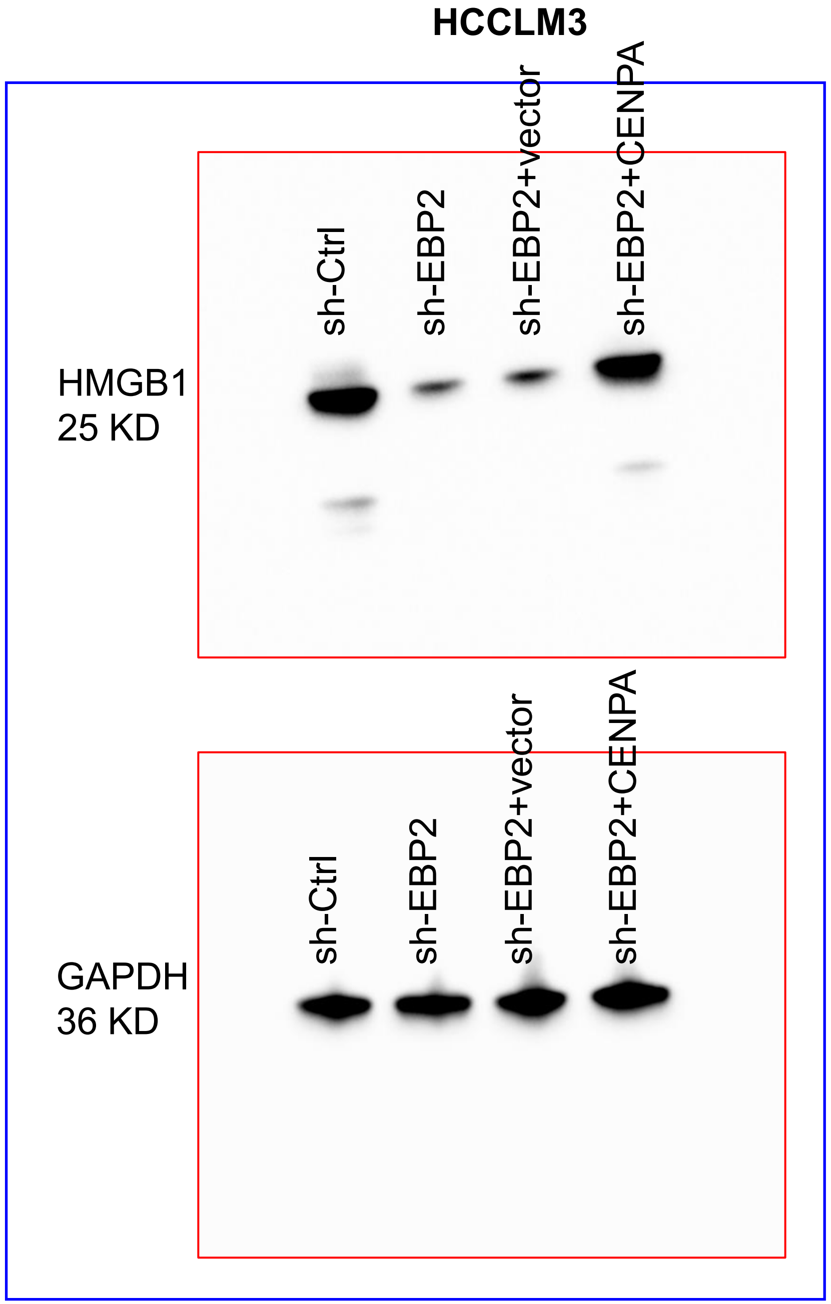

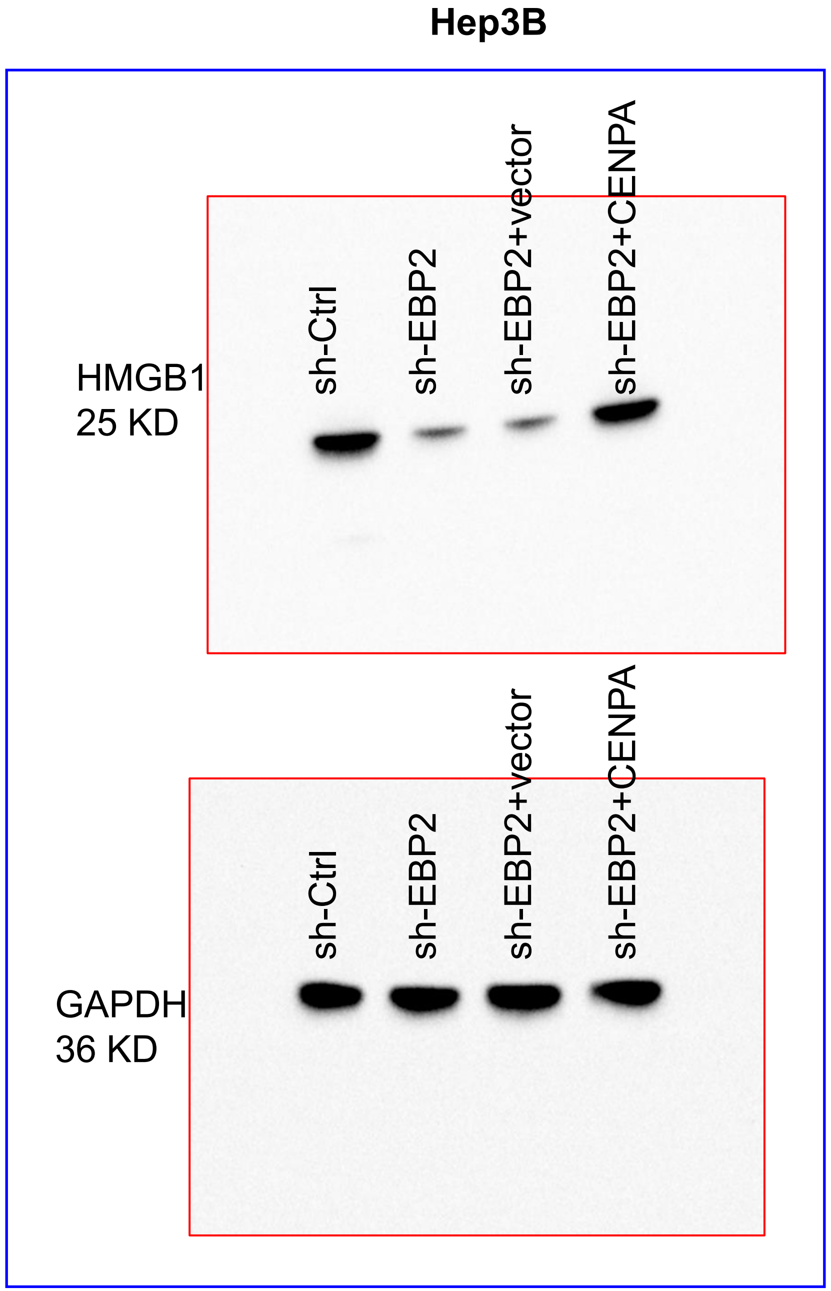

Raw data of WB for Figure 7G (upper panel in HCCLM3 and Hep3B cells)

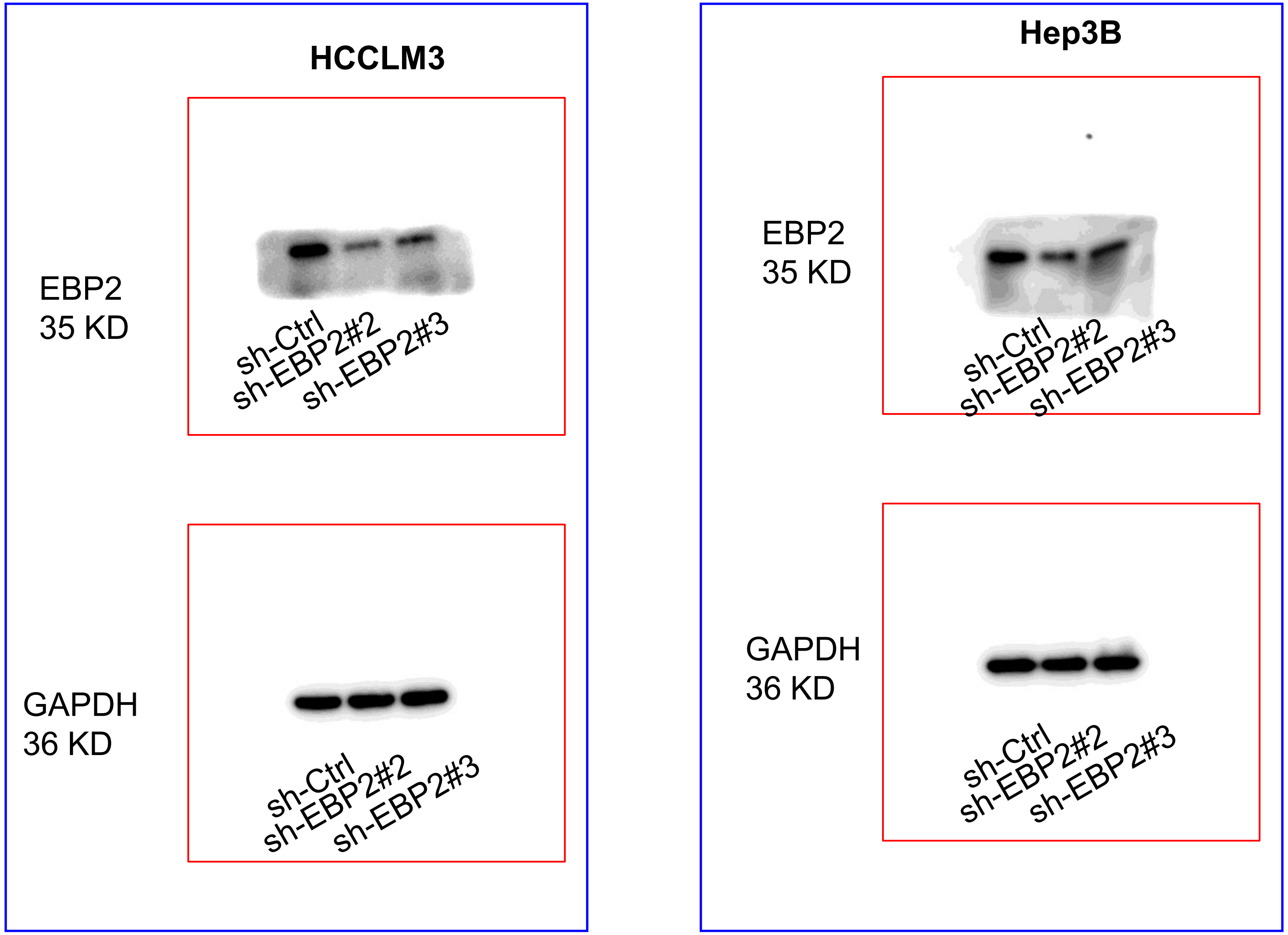

Raw data of WB for Figure S1D (upper panel in HCCLM3 and Hep3B cells)

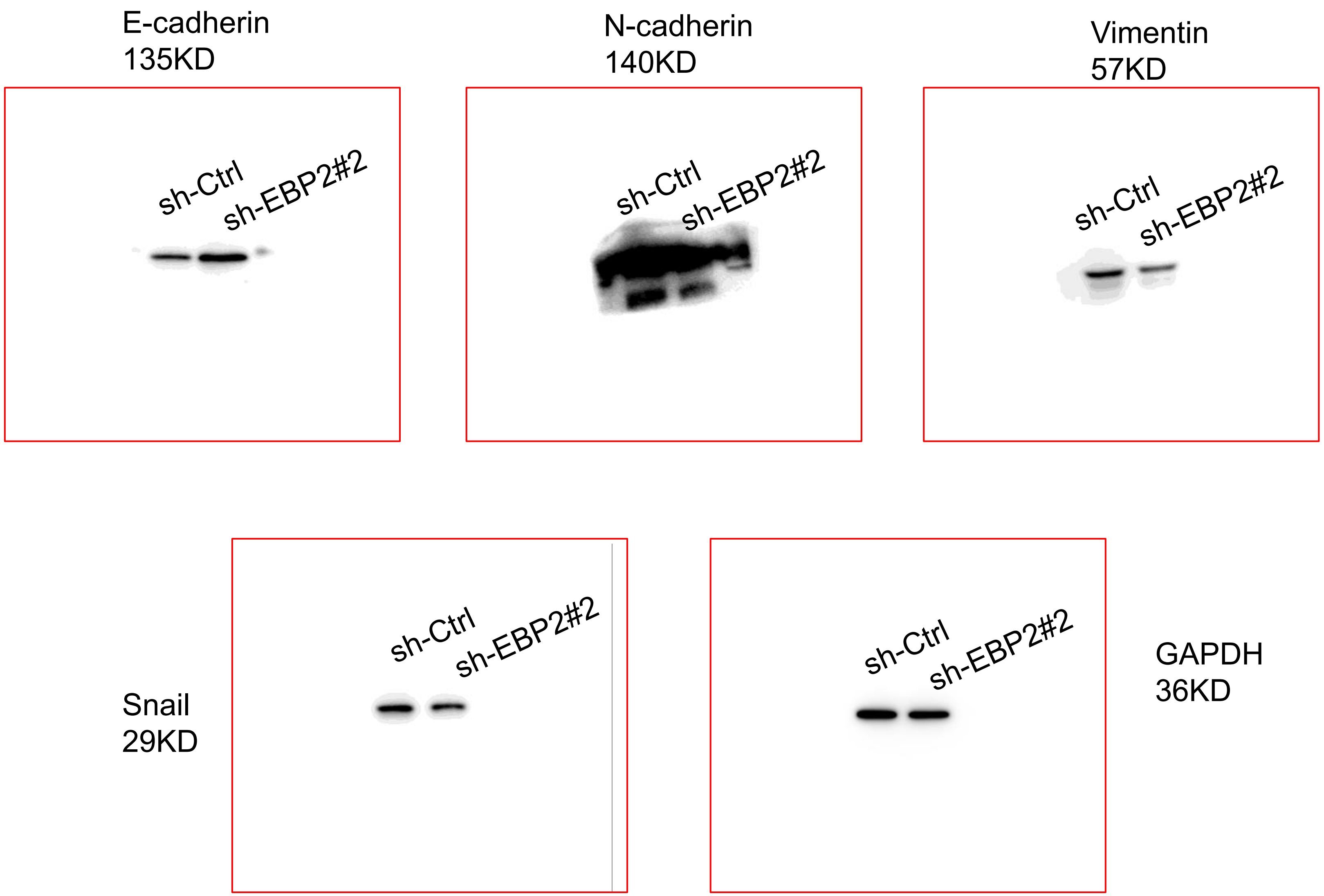

Raw data of WB for Figure S2 (upper panel in HCCLM3 cells)

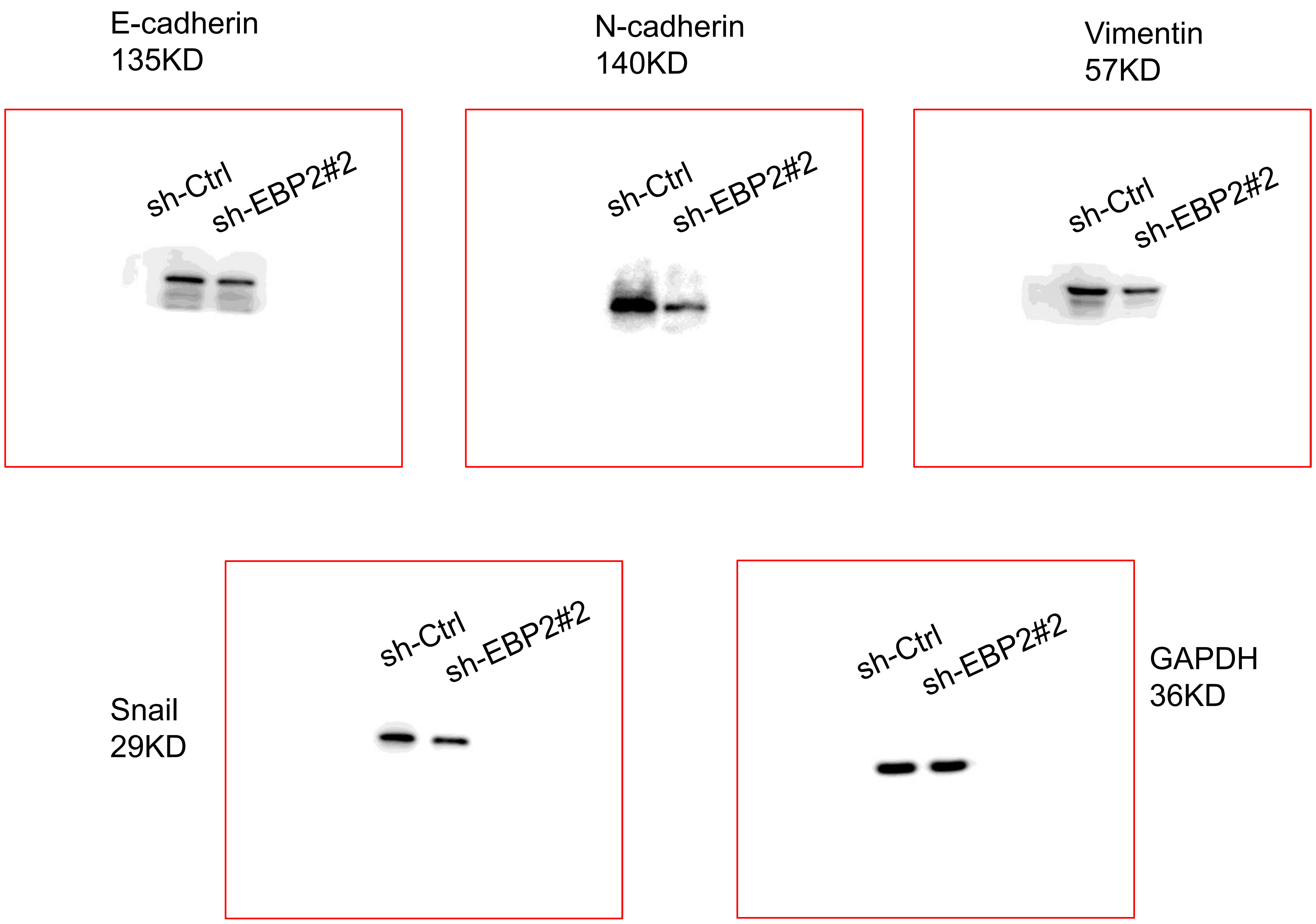

Raw data of WB for Figure S2 (upper panel in Hep3B cells)

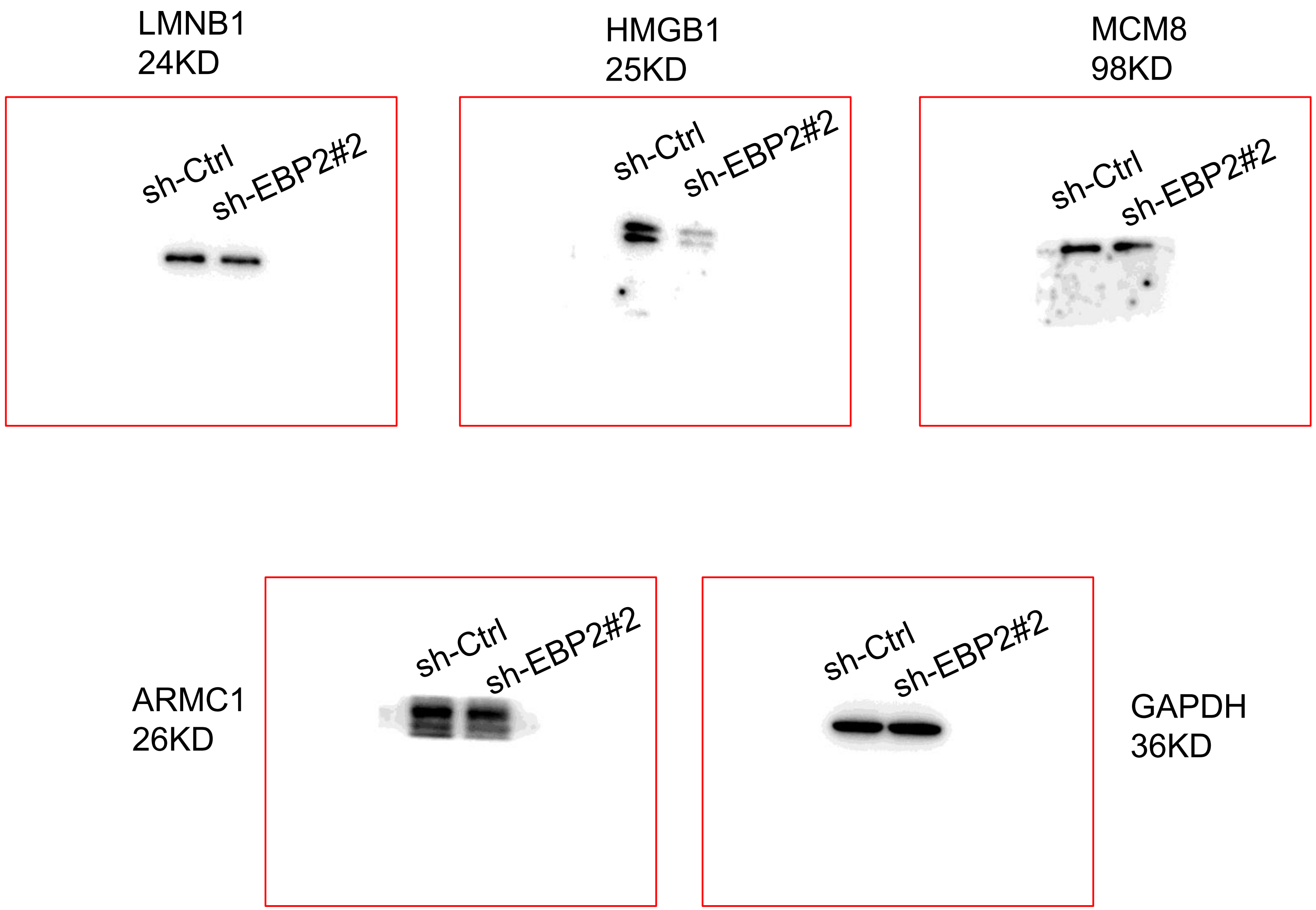

Raw data of WB for Figure S3B (upper panel in HCCLM3 cells)

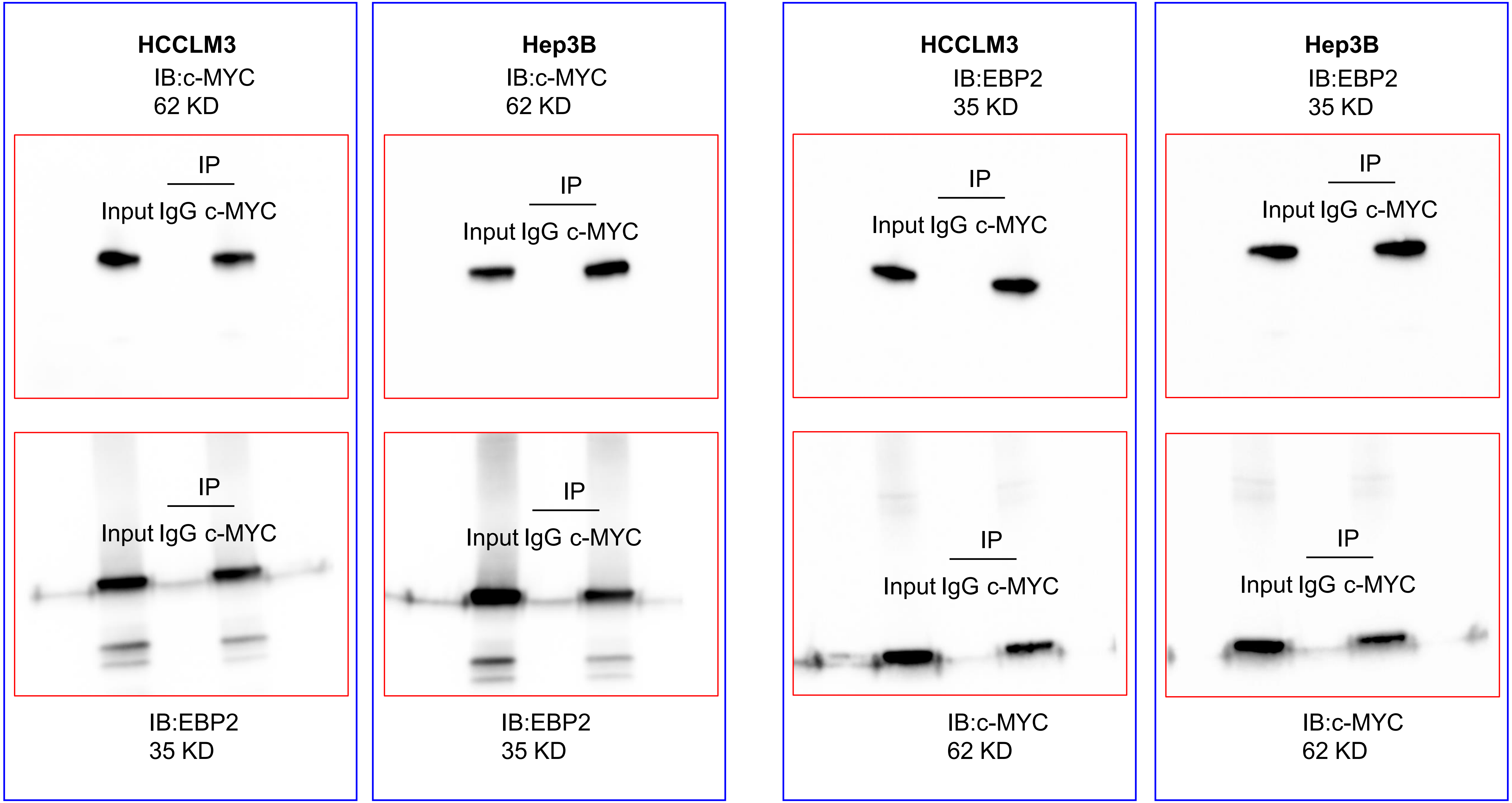

Raw data of WB for Figure S4A (upper panel in HCCLM3 and Hep3B cells)

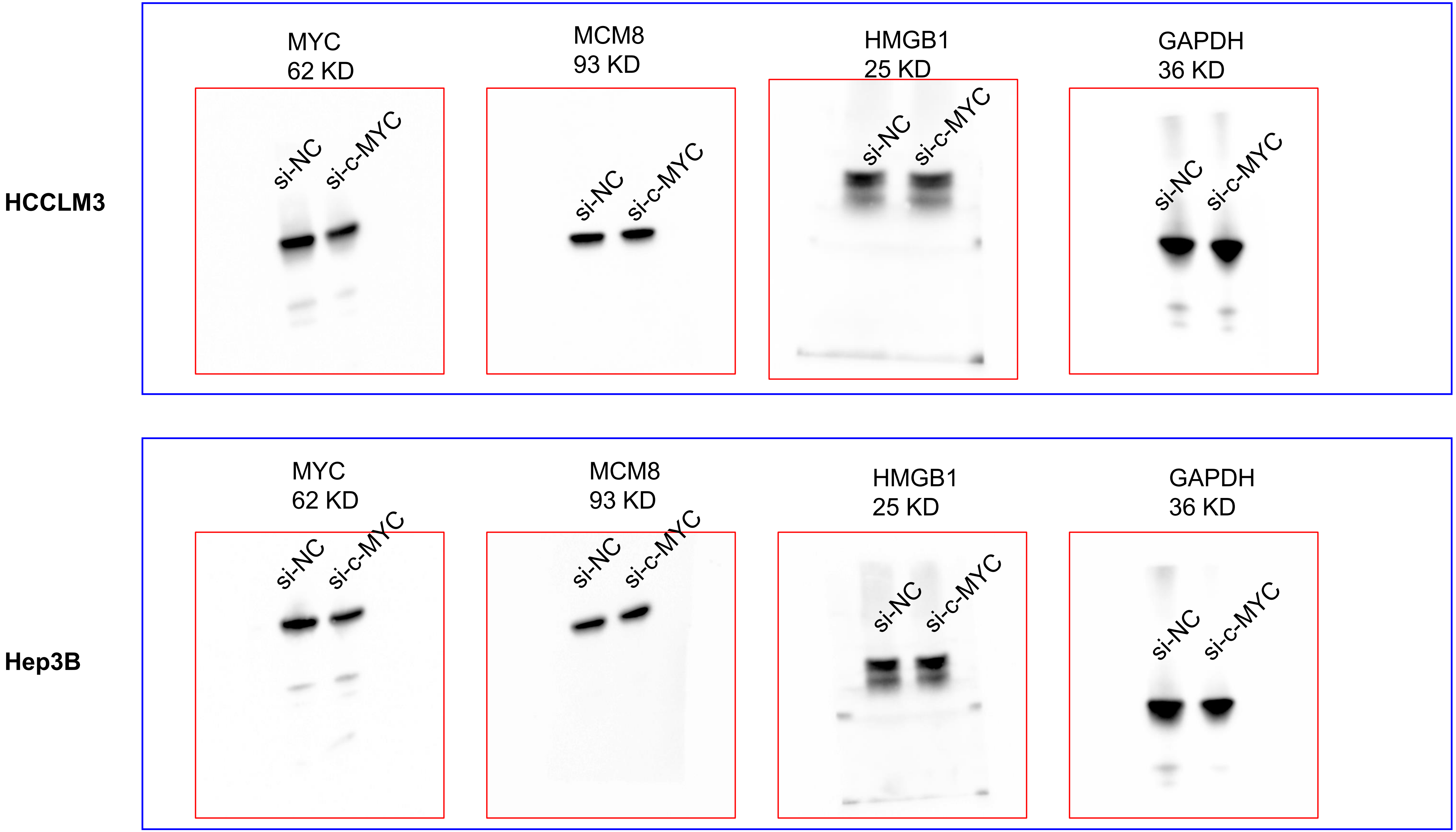

Raw data of WB for Figure S4C (upper panel in HCCLM3 and Hep3B cells)

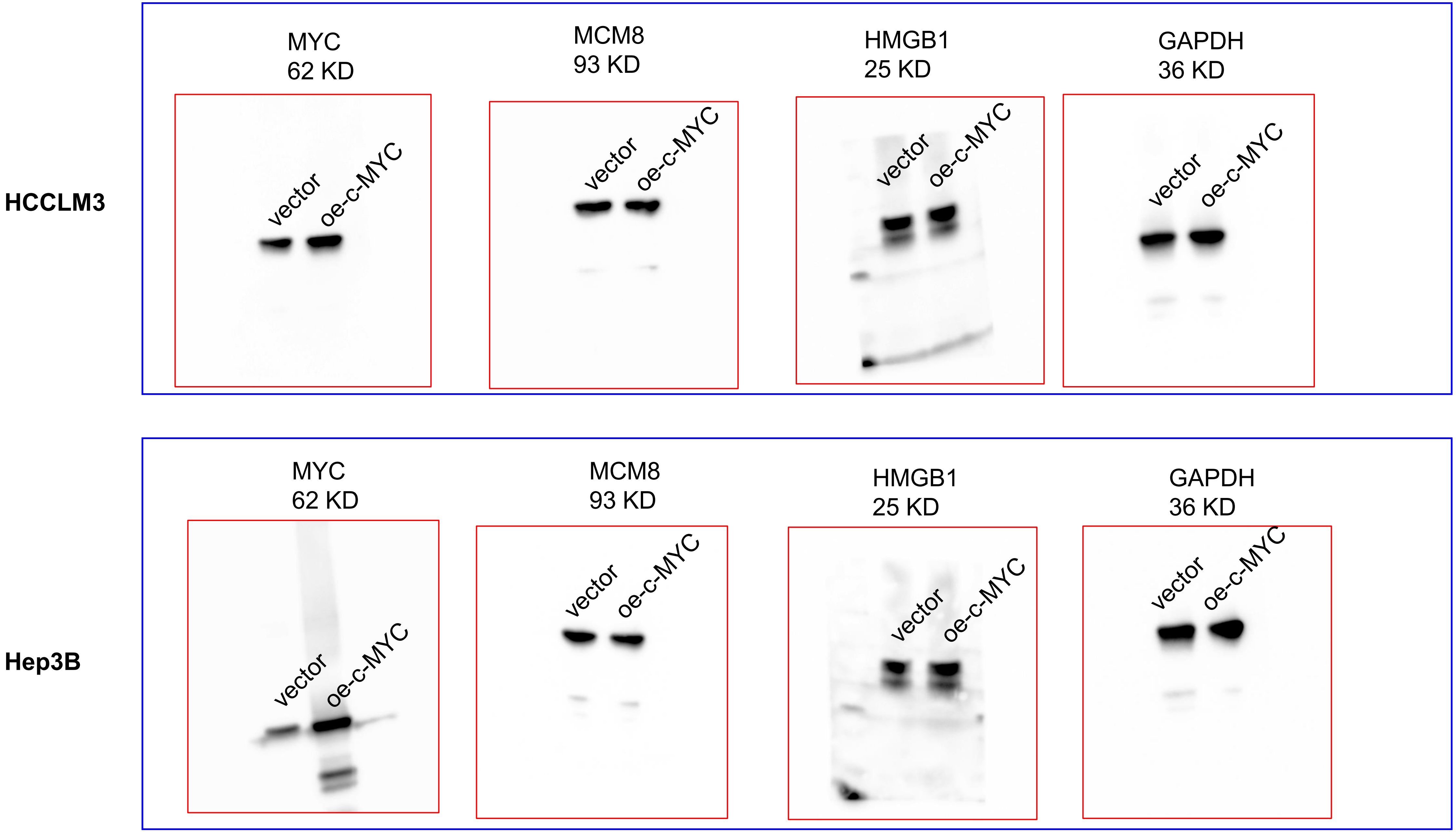

Raw data of WB for Figure S4C (upper panel in HCCLM3 and Hep3B cells)
