# Supplementary material for: EBNA1BP2 (EBP2) promotes the progression of hepatocellular carcinoma through upregulating the expression of MCM8 and HMGB1
Source: Cell Death Dis. 2026 Apr 4;17(1):596. doi: 10.1038/s41419-026-08671-8 (PMC13303876; doi:10.1038/s41419-026-08671-8)
Supplement: Supplementary file 2 — Supplementary Material [file 41419_2026_8671_MOESM2_ESM.pdf]

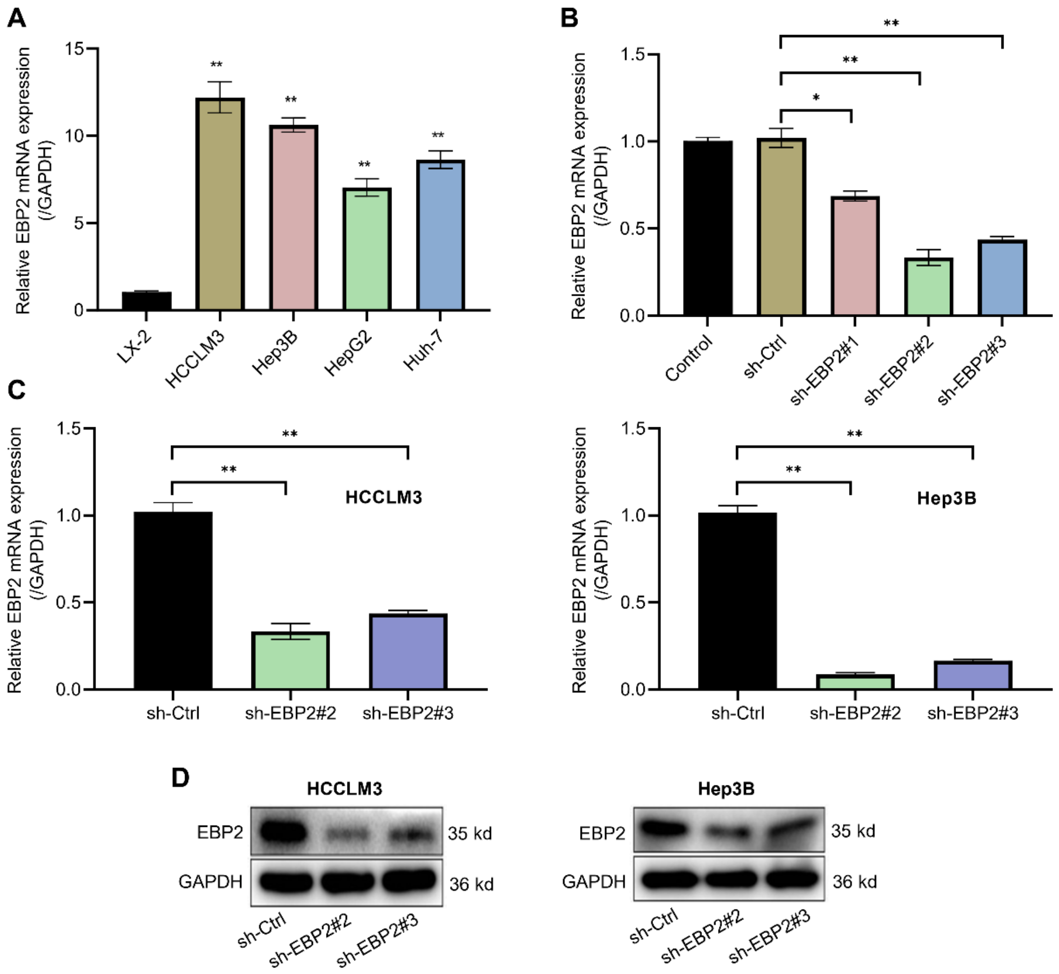

Figure S1. The expression level of EBP2 was up-regulated in HCC cell lines, while sh-EBP2#2 and sh-EBP2#3 effectively downregulated EBP2 expression. (A) The baseline expression level of EBP2 was detected in LX-2 cells and various HCC cell lines, including HCCLM3, Hep3B, HepG2, and huh-7. (B) The knockdown efficiencies of three different short hairpin RNAs (sh-RNAs) targeting EBP2 (sh-EBP2#1#2#3) was assessed by qPCR. (C, D) The knockdown efficiency of EBP2 was detected by qPCR and WB in HCCLM3 and Hep3B cells. Data were shown as mean ± SD ($n \geq 3$). * $P < 0.05$, ** $P < 0.01$.

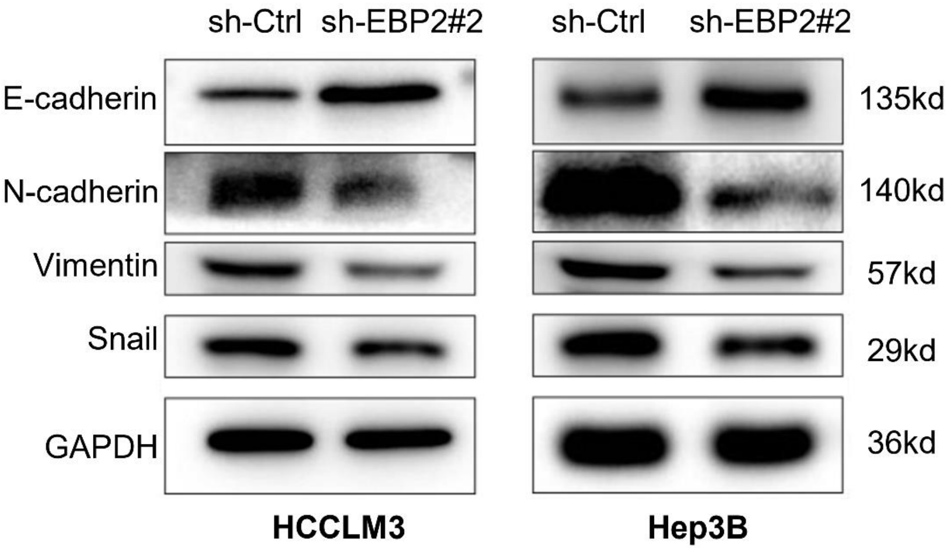

Figure S2. Knockdown of EBP2 suggests a reversal of EMT in HCC cells. The expression of EMT-related proteins including E-cadherin, N-cadherin, Vimentin and Snail was detected by WB in sh-Ctrl and sh-EBP2 groups of HCCLM3 and Hep3B cells.

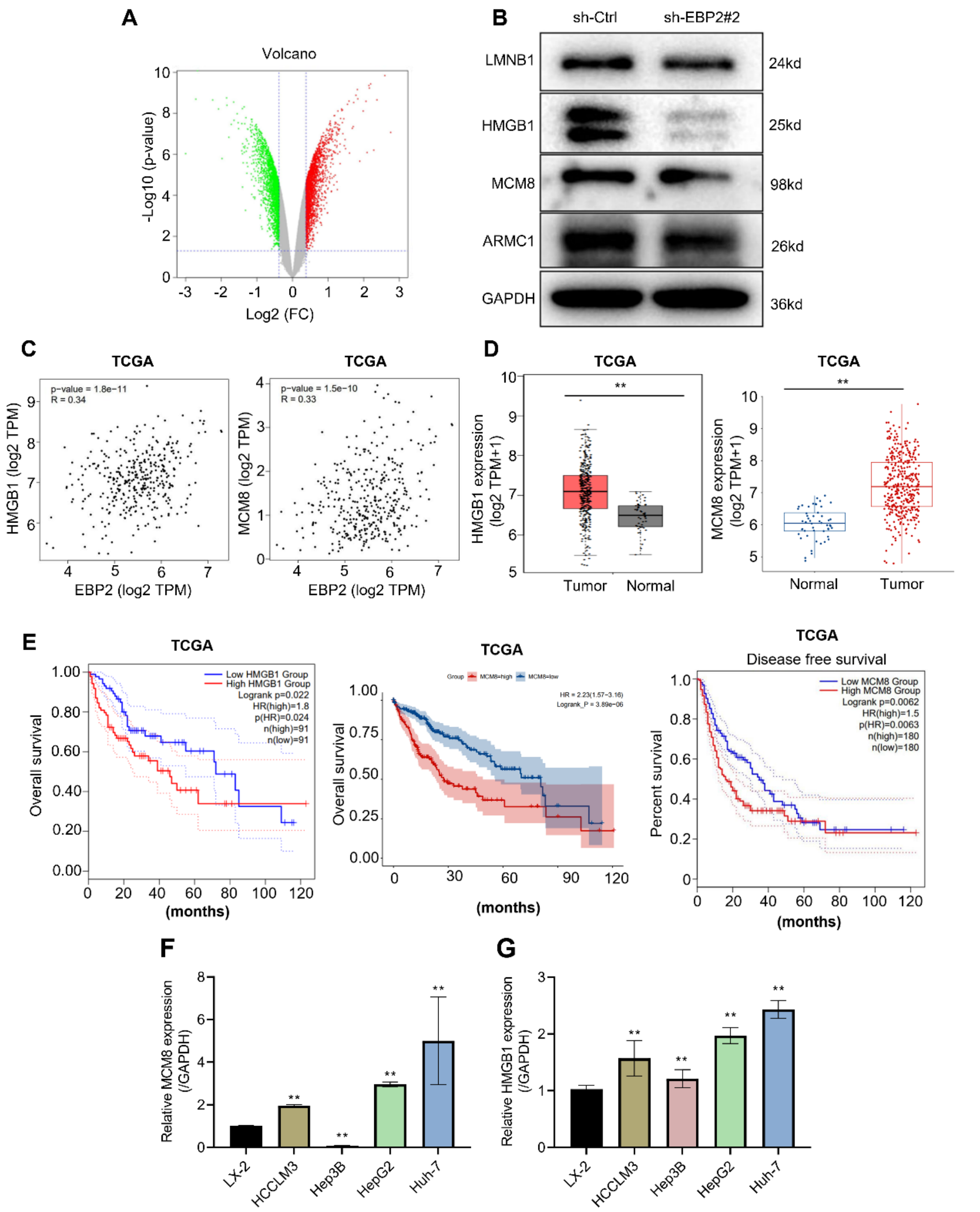

Figure S3. EBP2 may regulates MCM8 and HMGB1 transcription. (A) A genechip was used to identify significantly differentially expressed genes in sh-Ctrl and sh-EBP2 HCCLM3 cells. All the differentially expressed genes were drawn into the volcano plot. Red dots showed the significantly upregulated genes. Green dots showed the significantly downregulated genes. (B) Several candidates were further selected for WB verification in sh-Ctrl and sh-EBP2 groups of HCCLM3 cells. (C)

Correlation analysis of EBP2 with MCM8 and HMGB1 expression based on TCGA-LIHC data. (D) Expression levels of MCM8 and HMGB1 in tumor versus normal tissues based on TCGA data. (E) Based on the gene expression profiling data collected from TCGA dataset, the correlation between MCM8 and HMGB1 and overall survival of HCC patients was analyzed. (F) The baseline expression level of MCM8 and HMGB1 were detected in LX-2 cells and various HCC cell lines. Data were shown as mean ± SD ($n \geq 3$). ** $P < 0.01$.

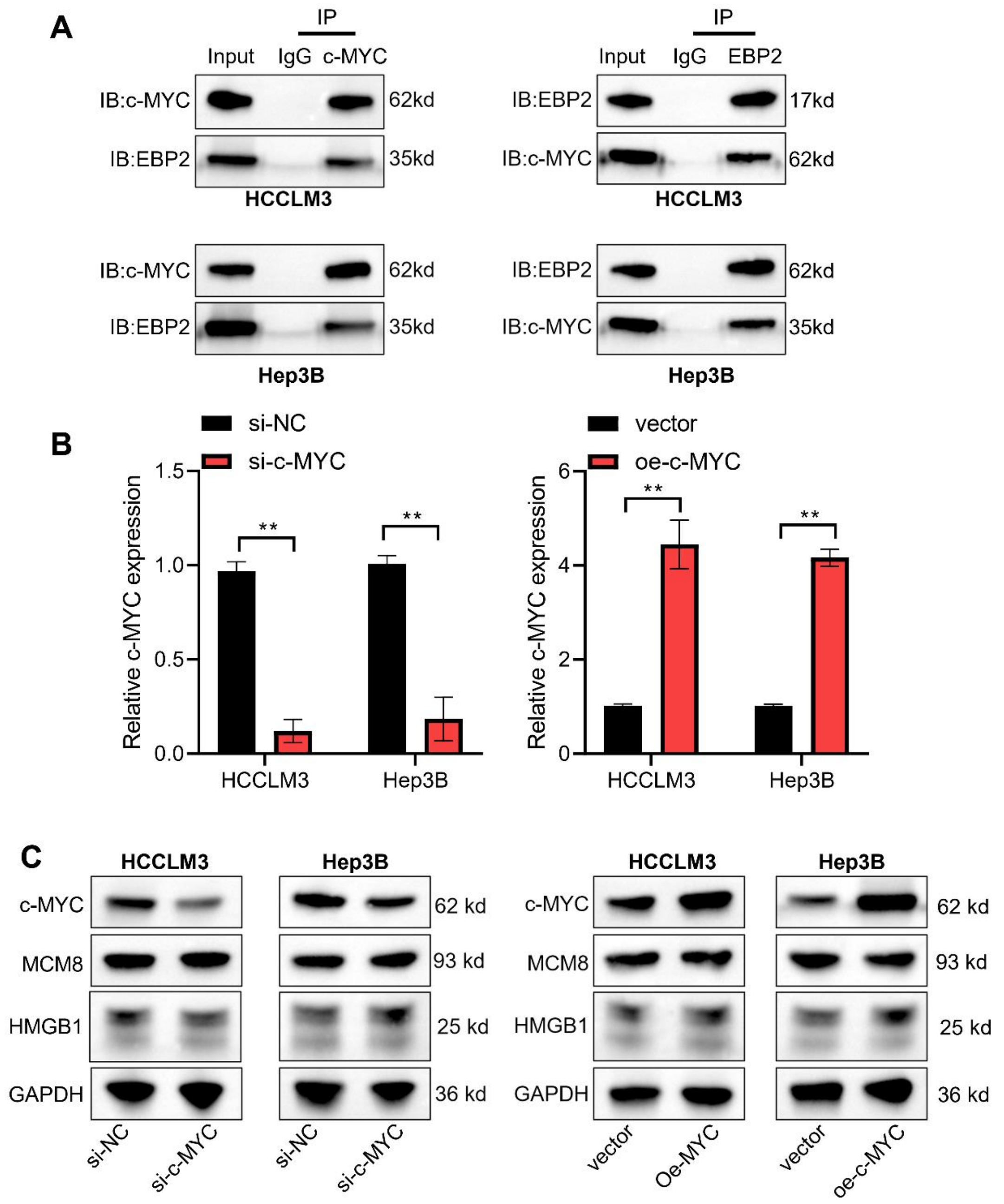

Figure S4. EBP2–MYC interaction fails to regulate MCM8 and HMGB1.

(A) co-IP confirms EBP2-MYC binding in HCC cells. (B) qPCR validates efficient MYC knockdown and overexpression in HCCLM3 and Hep3B cells. (C) WB shows that MYC depletion or overexpression does not alter MCM8 or HMGB1 levels. Data were shown as mean ± SD ($n \geq 3$). ** $P < 0.01$.

Table S1. Antibodies used in WB and IHC.

Primary antibodies	Dilution in WB	Source species	Company	Catalog No.
EBP2	1:500	Rabbit	bioss	bs-14495R
GAPDH	1:30000	Mouse	Proteintech	60004-1-lg
E-Cadherin	1:3000	Rabbit	Proteintech	20874-1-AP
N-Cadherin	1:1000	Rabbit	bioss	bs-1172R
Vimentin	1:2000	Rabbit	bioss	bs-8533R
SNAIL	1:1000	Rabbit	sigma	SAB1306281
CHD1L	1:3000	Rabbit	abcam	ab197019
HMGB1	1:500	Rabbit	Bioss	bs-20633R
MCM8	1:500	Rabbit	sigma	AV36668
NUDT21	1:1000	Rabbit	Proteintech	10322-1-AP
SSX2IP	1:500	Rabbit	Saier biolab	SRP09068
MCM8 (co-IP)	1:50/1:3000	Rabbit	Proteintech	16451-1-AP
CENPA (co-IP)	1:2000	Rabbit	Abcam	ab45694
**Primary antibodies**	**Dilution in IHC**	**Source species**	**Company**	**Catalog No.**
EBP2	1:100	Rabbit	bioss	bs-14495R
MCM8	1:100	Rabbit	Thermo	PA5-41325
Ki67	1/300	Rabbit	abcam	ab16667
**Secondary antibody**	**Dilution**		**Company**	**Catalog No.**
HRP Goat Anti-Rabbit IgG (WB)	1:3000		Beyotime	A0208
HRP Goat Anti-Mouse IgG (WB)	1:3000		Beyotime	A0216
HRP Goat Anti-Rabbit IgG (IHC)	1:200		Abcam	Ab111909

Table S2. Primers used in qPCR.

Gene	Forward primer sequence (5’-3’)	Reverse primer sequence (5’-3’)
GAPDH	TGACTTCAACAGCGACACCCA	CACCCTGTTGCTGTAGCCAAA
EBP2	CGGGATCTGGAATGGGTTGA	TGGTCCTTGTTCTGAGGTGCTG
HMGB1	CAGATGCTTCAGTCAACTTCTCAG	CCGCTTTTGCCATATCTTCA
FAM111B	CCAGACAATTCCCAGGATTAGA	TAGCATACCGCCTACCCAGA

PHF5A	ATCTTTTGCCGCAAGCAG	AGAGTGCAGGGACGCACATA
MCM8	ATGGCTTTTCTTTGTGCTGC	CCAGTCCATCGTAACTGTGAGA
SSX2IP	AGCTTTACAGAAGCCGCTATTC	TGAGTTCTGGTGGTCAAAGGTAG
LMNB2	GCAGCTCAAGAACAACTCGG	TTGGGCGTGAACTTGTAGGC
CHD1L	AACTCCCATCCAGAACAGCC	GCGTTGAATAAAATCTCCCACC
NUDT21	AAGCCTTGTTTGCAGTCCC	TCCATATCCTGGTGCATTGTC
ATG12	GTCTCCCCAGAAACAACCACC	AGGAGCAAAGGACTGATTCACATA
NOX1	CTTGGCTTAGGGATTCACGG	GGAGTCACGATCATCCCACAT

Table S3. Relationship between EBP2 expression and tumor characteristics in patients with HCC analyzed by Spearman rank correlation analysis.

Tumor characteristics	index	
T Infiltrate	Pearson correlation	0.278
	Significance (two tailed)	0.013
	n	79
Stage	Pearson correlation	0.312
	Significance (two tailed)	0.005
	n	79

Table S4. Expression patterns of MCM8 in HCC tissues and normal tissues revealed in immunohistochemistry analysis.

MCM8	Tumor tissue		Normal tissue	
expression	Cases	Percentage	Cases	Percentage
Low	34	50.0%	81	97.6%
High	34	50.0%	2	2.4%

Table S5. Expression patterns of HMGB1 in HCC tissues and normal tissues revealed in immunohistochemistry analysis.

HMGB1 expression	Tumor tissue		Normal tissue	
	Cases	Percentage	Cases	Percentage
Low	34	50.0%	77	92.7%
High	34	50.0%	6	7.3%

Table S6. Relationship between MCM8 expression and tumor characteristics in patients with HCC.

Features	No. of patients	MCM8 expression		*P* value
		low	high	
All patients	68	34	34	
Gender				0.327
Male	57	27	30	
Female	11	7	4	
Grade				<0.001
II	34	26	8	
III	33	8	25	
Stage				0.076
I	23	14	9	
II	29	15	14	
III	15	5	10	
IV	1	0	1	
T Infiltrate				0.115

T1	23	14	9	
T2	30	15	15	
T3	4	1	3	
T4	11	4	7	

Table S7. Relationship between HMGB1 expression and tumor characteristics in patients with HCC.

Features	No. of patients	HMGB1 expression		*P* value
		low	high	
All patients	68	34	34	
Gender				0.742
Male	57	28	29	
Female	11	6	5	
Grade				<0.001
II	34	27	7	
III	34	7	27	
Stage				0.028
I	23	17	6	
II	29	10	19	
III	15	7	8	
IV	1	0	1	
T Infiltrate				0.253
T1	23	15	8	
T2	30	14	16	
T3	4	1	3	

T4	11	4	7

Table S8. Relationship between MCM8 expression and tumor characteristics in patients with HCC analyzed by Spearman rank correlation analysis.

Tumor characteristics	index	
Grade	Pearson correlation	0.522
	Significance (two tailed)	<0.001
	n	67

Table S9. Relationship between HMGB1 expression and tumor characteristics in patients with HCC analyzed by Spearman rank correlation analysis.

Tumor characteristics	index	
Grade	Pearson correlation	0.559
	Significance (two tailed)	<0.001
	n	67
